# Emergence of Alternative Structures in Amyloid Beta 1-42 Monomeric Landscape by N-terminal Hexapeptide Amyloid Inhibitors

**DOI:** 10.1038/s41598-017-10212-5

**Published:** 2017-08-30

**Authors:** Srirupa Chakraborty, Payel Das

**Affiliations:** IBM Thomas J. Watson Research Center, Yorktown Heights, NY 10598 USA

## Abstract

Alzheimer’s disease (AD) is characterized by deposition of amyloid beta (Aβ) peptides into senile plaques in the brain. While most familial mutations are associated with early-onset AD, recent studies report the AD-protective nature of two genetic human Aβ variants, *i.e*. A2T and A2V, in the heterozygous state. The mixture of A2V Aβ1-6 (Aβ_6_) hexapeptide and WT Aβ1–42 (Αβ_42_) is also found neuroprotective. Motivated by these findings, in this study we investigate the effects of WT, A2V, and A2T Aβ_6_ hexapeptide binding on the monomeric WT Aβ_42_ landscape. For this purpose, we have performed extensive atomistic Replica Exchange Molecular Dynamics simulations, elucidating preferential binding of Aβ_42_ with the A2V and A2T hexapeptides compared to WT Aβ_6_. A notable reorganization of the Aβ_42_ landscape is revealed due to hexapeptide association, as manifested by lowering of transient interactions between the central and C-terminal hydrophobic patches. Concurrently, Aβ_6_-bound Aβ_42_ monomer exhibits alternative structural features that are strongly dependent on the hexapeptide sequence. For example, a central helix is more frequently populated within the A2T-bound monomer, while A2V-bound Aβ_42_ is often enhanced in overall disorder. Taken together, the present simulations offer novel molecular insights onto the effect of the N-terminal hexapeptide binding on the Aβ_42_ monomer structure, which might help in explaining their reported amyloid inhibition properties.

## Introduction

Alzheimer’s disease (AD), presently accounting for 60–70% of the 47.5 million dementia cases worldwide^1^, is symptomatic of progressive and irreversible loss of cognitive brain functions including memory, language skills, and spatiotemporal perception. AD is neuro-pathologically characterized by the aggregation of amyloid beta (Aβ) peptides into extracellular senile plaques, formation of intracellular neurofibrillary tangles, and subsequent neuronal degeneration^2, 3^. The causative Aβ peptide exists in varying lengths, Aβ_40_ and Aβ_42_ being the most abundant isoforms. Of these, Aβ_42_ is more aggregation-prone and toxic in nature^4^.

Solid-state (ss) NMR studies reveal that both Aβ_40_ and Aβ_42_ fibrils are composed of intermolecular stack of β-strands, termed as cross-β conformation, formed via self-complementary side-chain interfaces – so-called dry steric zipper^5^. In both Aβ_40_ and Aβ_42_ fibrils, residues around the central hydrophobic cluster (CHC) and the hydrophobic C-terminal region (CTR) form two parallel, in-register β-strands separated by a hydrophilic turn region^6, 7^. Structural polymorphism has been reported within Aβ fibrils, revealing alternative β-sheet arrangements, such as a triple β-motif^8^ or a double horse-shoe like structure composed of five β-strands^9^.

Aβ aggregation is initiated by a rate-limiting primary nucleation process, in which monomers self-associate into oligomeric ‘paranuclei’, followed by fibril assembly^10^. Oligomers can also be formed via a fibril-catalyzed secondary nucleation pathway from the monomeric peptides^11^. Soluble Aβ oligomers are reportedly more toxic than the fibrils and monomers, and are considered as pathological target for the treatment of AD^12, 13^. The neurotoxicity of those oligomers is correlated to their β-sheet content^14^ that increases with the order of polymerization^15^. Possible mechanism for Aβ oligomer toxicity includes direct association with membrane, resulting in membrane disruption and/or pore formation^16^.

**Figure 1:**
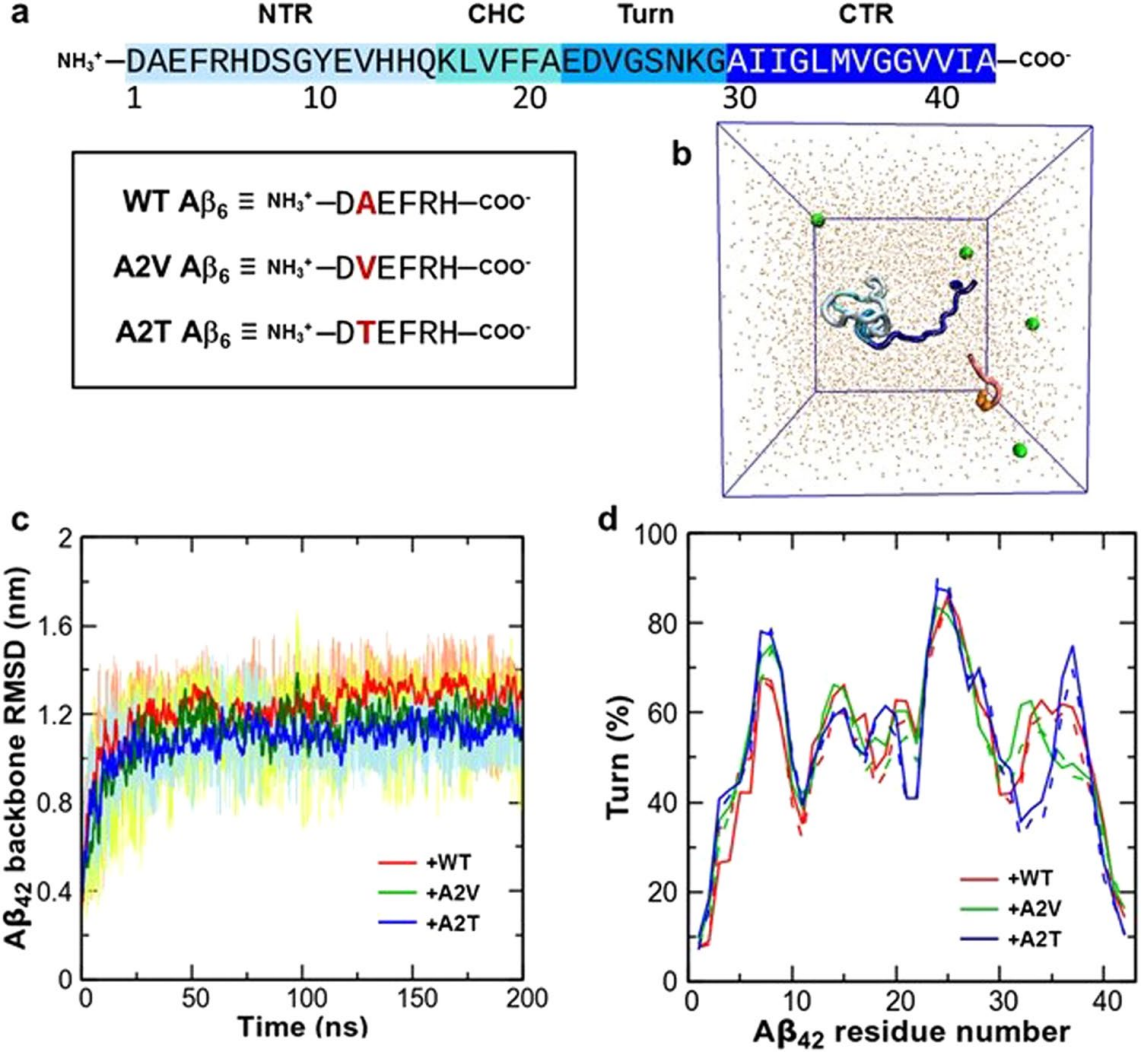
Simulation of Aβ_42_ with WT, A2V, or A2T Aβ_6_. (**a**) Amino acid sequence of Aβ_42_. Residues 1–16, 17–21, 22–29, and 30–42 of Aβ_42_ are denoted as NTR, CHC, turn and CTR, respectively. Color-code used to describe different regions of Aβ_42_ is also shown. Aβ_6_ derivatives are shown inside the black rectangle. Amino acid at position 2 of the hexapeptide is depicted in red. (**b**) The initial structure of the WT Aβ_42_ monomer and the A2T Aβ_6_ fragment immersed in water. Peptides are shown using cartoon representation. Aβ_42_ chain is color-coded according to Fig. 1a. Aβ_6_ is shown in red, and residue 2 is represented in orange using van der Waals (vdW) spheres. Water molecules (in brown) and sodium ions (in green) are shown as points and spheres respectively. (**c**) Backbone RMSD (in nm) of Aβ_42_, with respect to the initial peptide structure, as a function of simulation time (in ns) at 308.4 K. Moving averages of 1 ns are shown. The raw data (WT-bound: light brown, A2V-bound: light green, A2T-bound: light blue) are also plotted. (**d**) Residue-wise turn propensity (in %) at 308.4 K, using two different time-windows, 60–130 ns (solid line) and 60–200 ns (dashed line).

The monomer to oligomer transition is thought to be initiated by transient misfolding of the monomer to an aggregation-competent, hairpin-like conformation^17–22^. This structure is topologically similar to the peptide monomer conformation in fibrils, in which CHC and CTR are in direct contact, and residues 22–29 form a turn-like structure^19, 23, 24^ (hereafter referred as the turn region, see Fig. 1a). Designed Aβ sequences that fold into β-hairpin monomer conformation reportedly stabilize toxic, β-sheet rich oligomers and inhibit amyloid fibril formation^20^. However, translating the structural knowledge of the ordered amyloid fibrils to the Aβ monomer and pre-fibrillar, soluble oligomers remains non-trivial using conventional experiments, as those species are highly metastable, structurally heterogeneous, and disordered in nature^25, 26^. Aβ peptide belongs to the class of Intrinsically Disordered Peptides (IDP)^27^ that lack unique 3-dimentional structure, and instead exist as a dynamic ensemble of interconverting conformations. Molecular simulations at various resolutions^28–33^ have served as a useful means, particularly when combined with experiments, to characterize structure and binding of the disordered peptides.

The Aβ N-terminal region (NTR), that is primarily disordered in fibril structures^6, 7^, has long been relatively neglected in terms of its role in downstream events of AD. However, a number of familial mutations leading to aberrant monomer misfolding, aggregation, and related toxicity, have been identified at the NTR, such as the English (H6R) and the Tottori (D7N)^34^. Interestingly, a familial A2V mutation, while causing dementia in homozygous carriers, has been reported to provide protection in heterozygous carriers^35^. In a seminal whole-genome sequencing study on an Icelandic population, the rare A2T mutation was found to be AD protective and also offered protection from age-related cognitive decline in non-AD patients^36^. Experimental^37–41^ and simulation studies^42–44^ have revealed that, both A2V and A2T mutations differentially affect the Aβ monomer and oligomer structures, peptide aggregation kinetics, and associated toxicity. The protective nature of the WT/A2V(T) Aβ cross-interactions thus provides a natural path towards design of mechanism-based AD therapeutics. Along this line, Di Fede *et al*.^45^ recently showed that Aβ1-6_A2V_ in D-form directly interacts with WT Aβ_40/42_ and prevents amyloid fibril formation *in vivo*. Further testing of the hexapeptide tagged with the TAT sequence [Aβ1-6_A2V_TAT(D)] in a mouse model revealed alteration of oligomer size distribution, aggregation inhibition, and cerebral amyloid deposition^46^. However, little is known about the molecular mechanism underlying the inhibitory effect of those short NTR peptide fragments.

Given the importance of monomer misfolding in the amyloidogenesis pathway, it can be expected that binding of these Aβ-derived short inhibitor peptides results in remodeling of the full-length Aβ monomer landscape, thereby interfering with the subsequent aggregation. In this study, we take the first steps towards quantifying the effects of the binding of three different Aβ NTR hexapeptide (Aβ6) variants on the Aβ_42_ monomeric landscape. The hexapeptide variants studied are WT, A2V, and A2T Aβ_6_. For this purpose, we have performed large-scale, atomistically-detailed Replica Exchange Molecular Dynamics (REMD) simulations of the full-length peptide and hexapeptide variant in explicit water. In recent years, REMD has been extensively used as a tool to successfully sample conformational ensembles of amyloid proteins^43, 44, 47–49^. Our simulations reveal that these NTR-derivatives cause a substantial and sequence-dependent reconfiguration of the Aβ_42_ landscape. Taken together, this simulation study illustrates the potential of the NTR-based hexapeptides toward mechanism-based therapeutic design and calls for further extensive structural investigation, both computational and experimental.

## Results

### Simulating Aβ_42_ monomer in presence of WT, A2V, or A2T Aβ_6_ hexapeptide variant

The amino acid sequence of the Aβ_42_ monomer and the three Aβ_6_ NTR-derivatives studied here are shown in Fig. 1a. For brevity, we will refer these three Aβ_42_ + Aβ_6_ systems as WT-bound (or + WT), A2V-bound (or + A2V), and A2T-bound (or + A2T) henceforth. The simulated system comprising of the solvated full-length peptide monomer and hexapeptide variant is represented in Fig. 1b. To quantify the effect of Aβ_6_ binding on Aβ_42_ landscape, we compared the simulations of Aβ_42_ in presence of Aβ_6_ with earlier reported simulations of the free Aβ_42_ monomer^42^.

To ensure that the choice of the initial peptide conformation did not bias the results, we computed the root-mean-square deviation (RMSD) of the Aβ_42_ backbone from the initial peptide structure as a function of simulation time. Figure 1c depicts the backbone RMSD evolution of the 308.4 K replica as an example. The backbone RMSD reaches a value of 1.00 nm around 20 ns, then quickly equilibrates to ~1.20 nm by 50 ns, and fluctuates around this value (with a standard deviation of 0.12 nm) for the rest of the simulation. Based on this result, we consider that the REMD trajectories reached equilibration by around 50 ns, and the 60–200 ns portion of the trajectories was regarded as the production run. The production ensemble consists of structures extracted every 50 ps from the twelve replicas (276.0–308.4 K), resulting into 33,600 structures.

We further confirmed sampling convergence by comparing the residue-wise turn propensity of Aβ_42_ over the time intervals of 60–130 ns and 60–200 ns (Fig. 1d, also see Table S1). The overall turn propensity shows a mean value of ~50% for the hexapeptide-bound Aβ_42_ monomer, and is almost indistinguishable over the two time-intervals. Equilibration and convergence of the simulations were also tested in terms of additional structural properties (see Supplementary Information, Fig. S1, and Table S1). These results suggest that the production ensemble is not biased towards the initial conformation and has reasonably converged to a quasi-equilibrium state.

The radius of gyration (R_g_) distribution of the Aβ_42_ monomer, as estimated from the production ensemble (see Supplementary Fig. S2), reveals a mean value around 1.05 nm for the free Aβ_42_ and is unchanged upon hexapeptide binding. This value agrees well with the value of the hydrodynamic radius (0.9 nm) reported in fluorescence correlation spectroscopy measurements^50^ and R_g_ values reported in earlier REMD simulation studies^47, 51^. The estimated R_g_ value indicates a collapsed structure, as R_g_ scales as N^1/3^ (where N = 42)^52, 53^. We have also quantified the extent of disorder within the production ensemble by performing an RMSD-based clustering (see Supplementary Information). The results, as shown in Supplementary Fig. S2, imply that the top 50 clusters cumulatively represent ~90% of the total population for all systems. Thus, the Aβ_42_ peptide remains intrinsically disordered in presence of the hexapeptide variant, to the same extent as it is in its free state.

### Hexapeptide interaction modifies Aβ_42_ secondary structure profile

**Figure 2:**
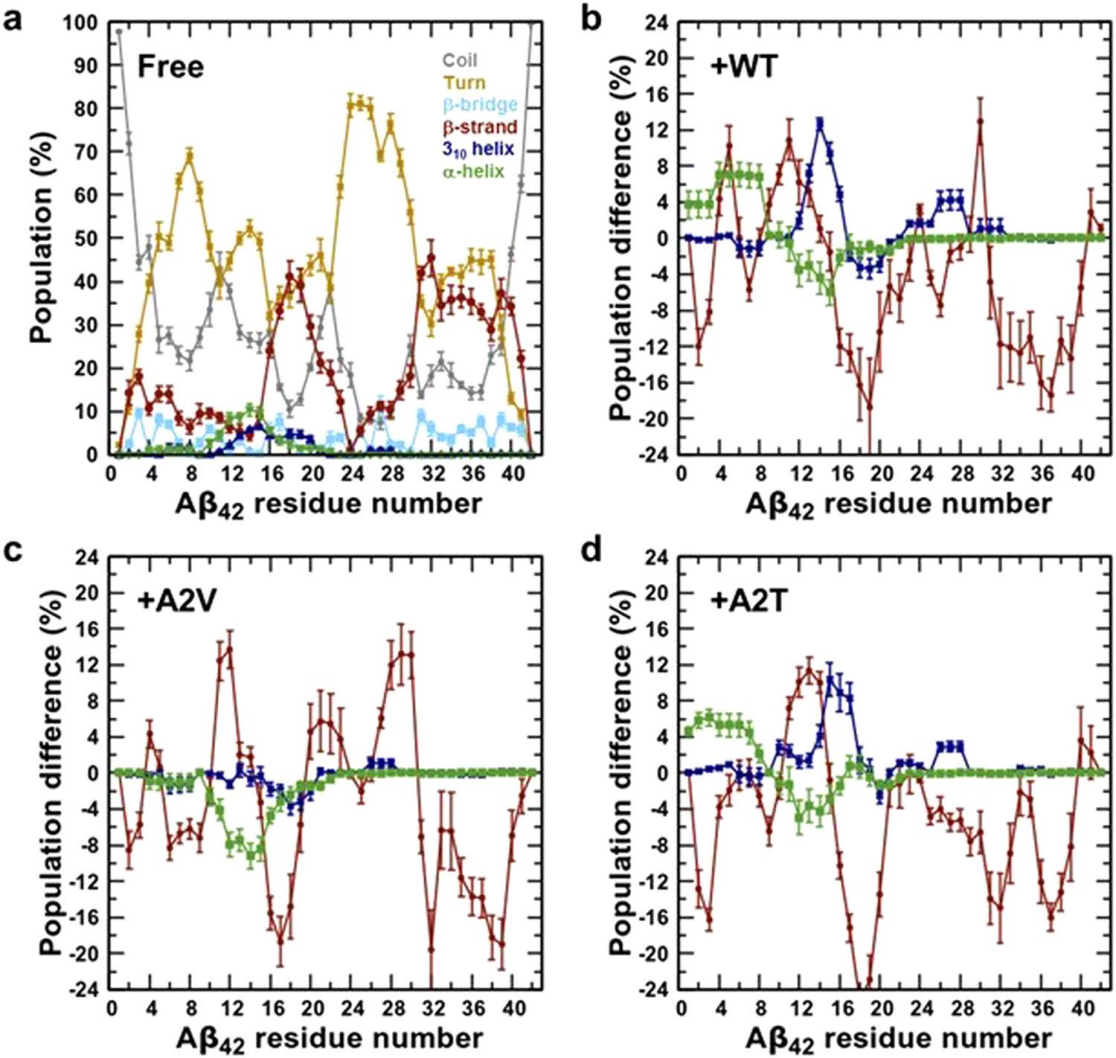
Secondary structural content of bound and free Aβ_42_. (**a**) Residue-wise population distribution (in %) of the secondary structural elements in free Αβ_42_ monomer. The values were estimated from our earlier published REMD study^42^. Standard errors of mean were estimated by dividing the production portion of replicas into four 35 ns long, non-overlapping blocks (see Methods). (**b**–**d**) Differences (in percentage) in residue-wise β-strand, 3_10_-helix, and α-helix propensities between bound and free Aβ_42_. Positive values indicate higher occurrence in the hexapeptide-bound system, and vice versa. (**b**) WT-bound, (**c**) A2V-bound, and (**d**) A2T-bound.

Figure 2 shows the ensemble-averaged, residue-wise population of the secondary structure elements within the free Aβ_42_ ensemble. The standard error values were obtained from standard deviations estimated by dividing the simulation data into four 35 ns long, non-overlapping blocks between 60–200 ns. The calculated standard error values are negligible, indicating statistical significance of the values reported (see Model and Methods in Supplementary Information). A notable feature of the secondary structure profile of free Aβ_42_, is a >20% β-strand propensity around the CHC (residues 17–21) and the CTR (residues 30–41). The residue-wise secondary structure distributions of hexapeptide-bound Aβ_42_ can be found in Supplementary Fig. S3. The overall disordered nature of Aβ_42_ in all systems is illustrated, as coil and turn population together account for 70–75% of secondary structure. Hexapeptide binding in general results in considerable lowering of the CHC and CTR β-strand propensity (at least by 10% in majority of those residues, see Fig. 2b–d), when compared to free Aβ_42_. The β-strand tendency reduction is most prominent in the CHC residues. In contrast, the preCHC region (residues 11–16) exhibits enhanced β-strand tendency in all three hexapeptide-bound Aβ_42_ systems. β-strand formation is also noticed around residues 6 and 29 in WT-bound Aβ_42_ and residues 28–30 in A2V-bound Aβ_42_. The first 17 residues appear more helix-rich in the WT-bound and A2T-bound peptide (Fig. 2b,d). On the other hand, the helicity becomes almost negligible due to A2V hexapeptide binding.

The short peptide mainly populates coil structure (≥60%) in all three variant forms (Supplementary Fig. S4). Interestingly, the A2V hexapeptide shows notable (~15%) propensity to form β-strand around residues 2 and 3, when compared to the two other variants.

### Wild-type CHC-CTR interaction is reduced due to Aβ_6_ binding

**Figure 3:**
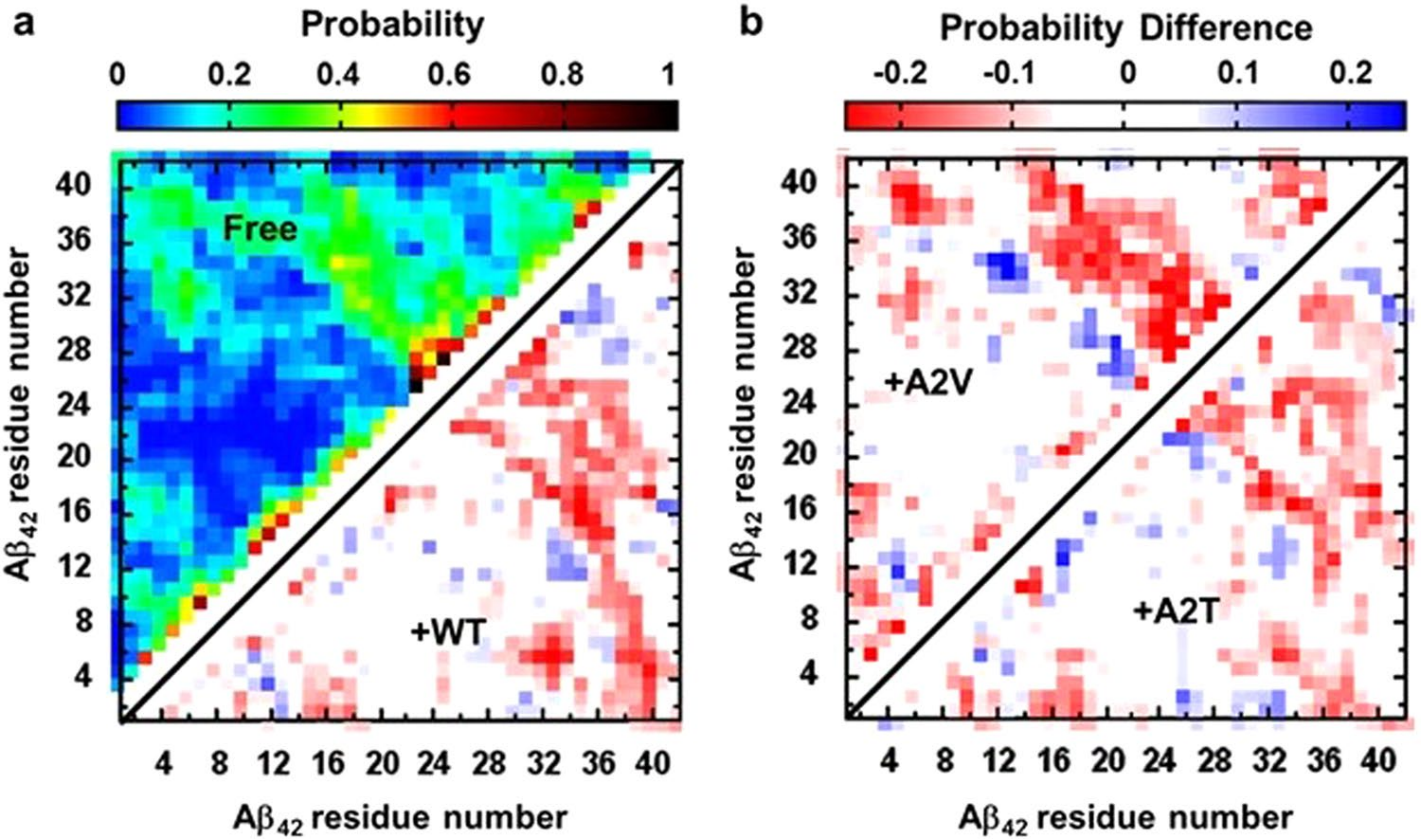
Characterization of Aβ_42_ tertiary structure. (**a**) Ensemble-averaged intramolecular C_α_ contact map of the free Aβ_42_ monomer^42^ (upper triangle). Only non-sequential contacts, *i.e*., |i–j| ≥ 3, are considered. Arithmetic difference between the contact probabilities of hexapeptide-bound and free Aβ_42_ are also plotted (a, lower triangle: WT-bound; b, upper triangle: A2V-bound; b, lower triangle: A2T-bound). Color-scales used for contact probability and contact probability differences (bound minus free) are shown on top of the figures.

Next, we compare the intramolecular contact map of the bound Aβ_42_ with that of the free peptide, to reveal the effect of short peptide binding on the Aβ_42_ tertiary structure (see Fig. 3 and Supplementary Fig. S5). It should be noted that all long-range (|i-j| > 8) contacts observed are transiently populated (with a ~12% average probability of formation) within the unbound Aβ_42_ ensemble, in accordance with its intrinsically disordered nature. Noticeable long-range interactions are: (i) a set of anti-parallel, hairpin interactions between the CHC and CTR residues, (ii) a second, less-extensive set of hairpin interactions within CTR residues 30–41, and (iii) contacts between two termini. The observed β-strand proclivity of the CHC and CTR residues, the dominant (>70%) turn tendency of residues 24–28, and the CHC-CTR tertiary interactions, together suggest transient population of a CHC-CTR β-hairpin monomer. Such transiently formed, extended hairpin-like monomer structures are believed to be aggregation-prone in nature, therefore triggering the formation of soluble, toxic oligomers and subsequent polymerization^20, 23, 24^.

Interestingly, the Aβ_42_ tertiary contact map reveals significant changes due to hexapeptide binding. Only the contacts showing a probability difference value higher than 0.06 were considered for this analysis. First of all, hexapeptide binding induces a marked ≥20% reduction in the CHC-CTR interaction (Fig. 3). At the same time, the D23-K28 salt-bridge frequency decreases from 13% in free Aβ_42_ to 8–9% in the bound peptide. These findings together suggest that hexapeptide binding reduces the transient folding into an aggregation-prone β-hairpin structure, of which CHC-CTR contacts and D23-K28 salt-bridge are key features^17, 18, 21, 54^. Additionally, interactions of extreme NTR with rest of the protein generally decrease in the bound Aβ_42_. On the other hand, enhanced interaction between the preCHC residues 12–16 and the CTR residues 30–35 is observed, following the order: A2T-bound (12%) <WT-bound (16%) <A2V-bound (26%). Resembling what was seen in secondary structure analysis (Fig. 2), the tertiary structural profile of the WT-bound and A2T-bound Aβ_42_ appear similar to each other. For example, the hairpin contacts within CTR are consistently present in the WT-bound and A2T-bound Aβ_42_ (to the same or increased extent as found in free Aβ_42_), but decreases upon A2V hexapeptide binding. Enhanced binding between the CHC region and residues 24–30 is noticed in the A2V-bound system (Fig. 3b). The strong β-strand propensity in those residues combined with enhanced turn tendency of residues 24–27 consistently suggest formation of a register-shifted β-hairpin in the A2V-bound Aβ_42_ (see Fig. 2 and Supplementary Fig. S3).

### A2V and A2T substitutions favor Aβ_42_-Aβ_6_ binding

**Figure 4:**
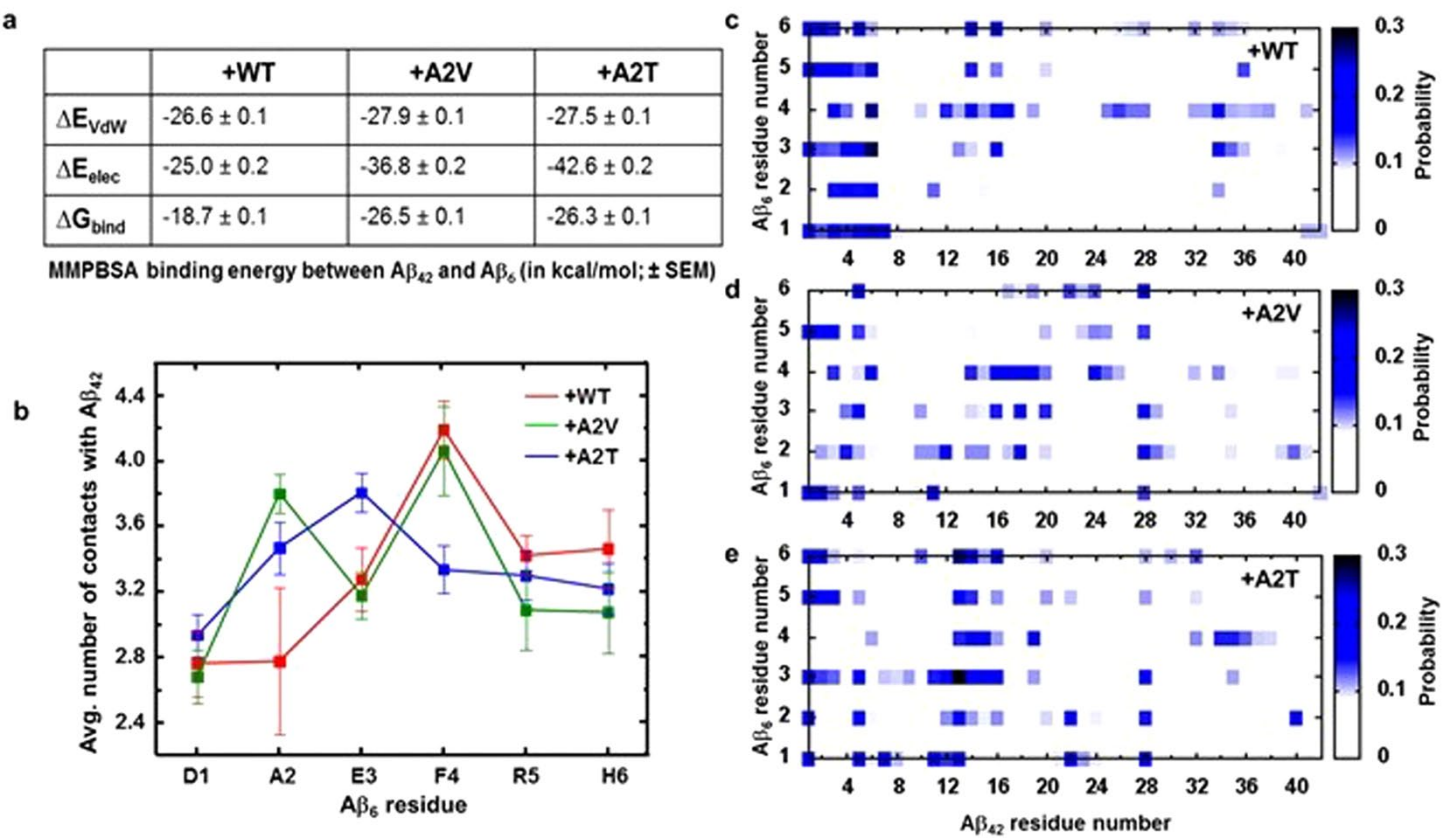
Intermolecular binding analysis. (**a**) Interpeptide binding free energy estimation, as estimated using the MM-PBSA method. Van der Waals, electrostatic, and total binding energy values (±standard errors of mean) are reported in kcal/mol. (**b**) Average number of Aβ_42_ heavy-atom contacts per residue across the hexapeptide sequence. Standard error values were estimated by dividing the production data into four 35 ns long blocks (see Methods). (**c**–**e**) Quaternary heavy-atom contact probability maps: (**c**) WT-bound, (**d**) A2V-bound, and (**e**) A2T-bound systems.

Binding free energy (∆G_bind_) between Aβ_6_ and Aβ_42_ was calculated by using the Molecular Mechanics - Poisson-Boltzmann Surface Area (MM-PBSA)^55^ method (Fig. 4a). Both electrostatic (∆E_elec_) and Van der Waals (∆E_VdW_) terms equally contribute to the WT Aβ_6_ – Aβ_42_ binding energy. Using WT-hexapeptide binding as reference, it was observed that binding is preferred upon A2V and A2T mutation. While ∆E_VdW_ remains the same (~−27 kcal/mol), ∆E_elec_ decreases from −25 kcal/mol to −37 kcal/mol in A2V-bound and −43 kcal/mol in A2T-bound Aβ_42_, indicating a strengthening of the interpeptide electrostatic interactions. Interestingly, SPR experiments also indicated more favorable binding of the 1–6 A2V peptide with Aβ_40_, when compared to the WT hexapeptide^45^. A comprehensive breakdown of the constituent MM-PBSA energy terms is given in Supplementary Table S2.

Figure 4b illustrates the average number of heavy-atom contacts with the full-length peptide across the hexapeptide residues. This analysis suggests average ~3.3 interpeptide heavy-atom contact formation per hexapeptide residue for all three Aβ_6_ variants. Figure 4b further indicates that, a valine or threonine at position 2 of Aβ_6_ interacts more frequently than an alanine. This frequency increase is stemming from the preferential interaction with Aβ_42_ residues 12–22, 28, and 40, as suggested by the interpeptide contact maps shown in Fig. 4c–e. A threonine at positon 2 of Aβ_6_ also allows E3 to be more interacting, especially with the preCHC residues 11–16. On the other hand, the probability of contact formation with aromatic F4 residue from Aβ_6_ is enhanced, when the hexapeptide variant is more hydrophobic in nature (WT or A2V). In those scenarios, the contacting Aβ_42_ residues are 3–6, 14–17, and 24–27 (Fig. 4c and d). The primary mode of hexapeptide binding involves NTR Aβ_42_ residues, irrespective of the Aβ_6_ variant sequence studied here (Fig. 4c–e). Residues 1–7 of Aβ_42_ exhibit ~12% probability of contact formation with A2V and A2T Aβ_6_, and ~17% with WT hexapeptide (Fig. 4c). Additional contacting Aβ_42_ residues are residues 12–17 and 34 with WT Aβ_6_ (Fig. 4c), CHC residues with A2V Aβ_6_ (Fig. 4d), and preCHC residues 11–16 with A2T Aβ_6_ (Fig. 4e). Overall, the A2V and A2T variants are more efficient in sequestering the central hydrophobic core and binding to K28, when compared to WT Aβ_6._ Thus, A2V and A2T Aβ_6_ appear stronger candidates for disrupting the CHC-CTR interaction and central salt bridge formation^17, 54^, which are crucial components of the β-hairpin structure found in oligomers and fibrils.

### Alternative structures are stabilized on the hexapeptide-bound Aβ_42_ landscape

**Figure 5:**
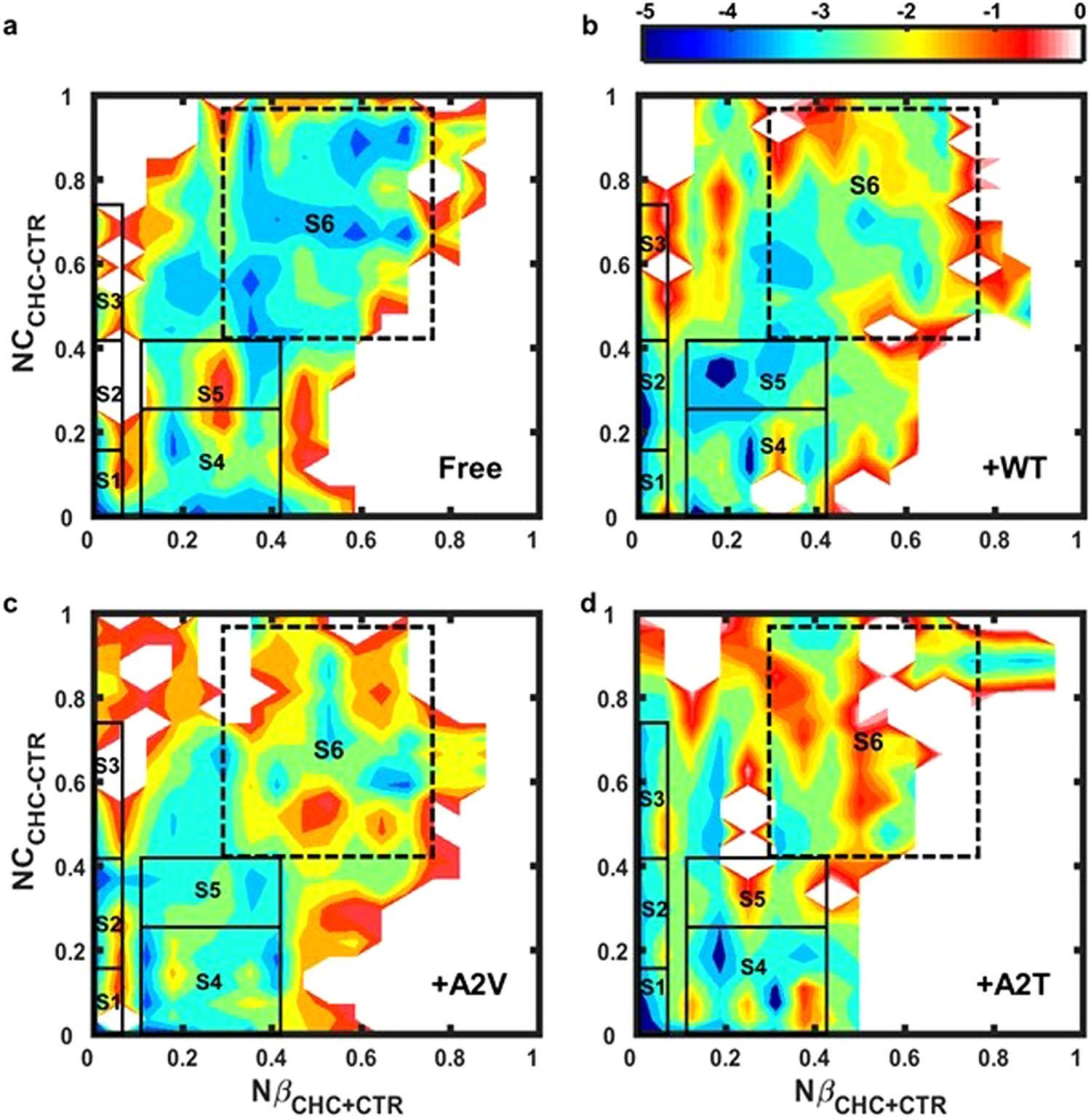
Bound and free Aβ_42_ monomeric landscapes. (**a–d**) 2D potential of mean force (PMF, in kcal/mol) plots as a function of the number of residues in CHC and CTR region that are in β-strand conformation (Nβ_CHC+CTR_), and the number of C_α_ contacts between CHC and CTR (NC_CHC-CTR_), both normalized to one. (**a**) free monomer, (**b**) WT-bound, (**c**) A2V-bound, and (**d**) A2T-bound systems. Solid black rectangles denote the S1–S5 states that are more frequently populated upon hexapeptide binding. The S6 sub-population (dashed black rectangle) corresponds to the CHC-CTR hairpin-rich region.

To further disseminate the changes in the Aβ_42_ monomeric landscape due to hexapeptide binding, we have estimated a two-dimensional Potential of Mean Force (PMF, see Methods and Fig. 5). For this purpose, the following two reaction coordinates were defined: (i) the number of residues in CHC and CTR region that are in β-strand conformation, Nβ_CHC+CTR_; and (ii) the number of C_α_ contacts between CHC and CTR, NC_CHC-CTR_; both normalized to one. This choice was motivated by the secondary and tertiary structural changes resulting from short peptide binding (Figs 2 and 3). Interestingly, while the full-length monomer in all four systems (three Aβ_6_-bound and free Aβ_42_) sampled similar regions of the energy landscape, the population distribution was substantially affected upon hexapeptide binding (Fig. 5). This observation led to further investigation of six regions on the PMF plot, S1 to S6 (see Fig. 5, Table S3 and Methods). The population distribution within these regions is shown in Table 1 for all systems, suggesting that the S1–S6 populations together represent 80–90% of the total ensemble in each case. The free Aβ_42_ monomer frequently (~47%) visits the S6 region that corresponds to high values of NC_CHC-CTR_ and Nβ_CHC+CTR_, indicating possible CHC-CTR β-hairpin population. However, a RMSD-based clustering of the S6 sub-population reveals high structural diversity even within this region. Only those structures with Nβ_CHC+CTR_ > 0.6 consistently show a single or a double hairpin motif comprised of CHC and CTR residues (Supplementary Fig. S6). Binding to Aβ_6_ variant dramatically reduces S6 population, e.g. to 21% in WT-bound, to 22% in A2V-bound, and to 13% in A2T-bound Aβ_42_ (Table 1), indicating that Aβ_6_ binding inhibits CHC-CTR hairpin interaction that is thought to be crucial for aggregation nucleation and toxicity^18, 20^.

**Table 1:** Percentage population (mean ± standard error) of the low PMF energy states.

	S1	S2	S3	S4	S5	S6	Others
Free Aβ _42_	11.6 ± 4.7	6.4 ± 0.7	2.6 ± 1.2	13.3 ± 1.5	5.7 ± 1.2	47.0 ± 3.8	13.4 ± 1.8
+WT	9.3 ± 3.7 (F4, A2)	*18.0* ± *2.1* (E3, H6)	2.6 ± 0.6 (A2, E3)	*18.3* ± *2.8* (R5, F4)	*16.5* ± *2.7* (R5, F4)	21.0 ± 3.7 (F4, H6)	14.3 ± 1.9
+A2V	*15.2* ± *2.7* (F4, V2)	*12.8* ± *0.7* (V2, E3)	4.4 ± 1.0 (F4, V2)	*23.1* ± *1.9* (F4, V2)	*11.6* ± *1.2* (V2, D1)	21.9 ± 4.6 (F4, R5)	11.0 ± 1.7
+A2T	*22.5* ± *1.8* (H6, T2)	6.9 ± 2.0 (T2, E3)	*11.1* ± *2.5* (E3, H6)	*20.0* ± *1.4* (R5, F4)	7.3 ± 1.0 (R5, E3)	12.9 ± 2.8 (H6, E3)	19.3 ± 1.1

The regions on the PMF landscape, which individually represents at least 10% of the total population, are given in italics and further considered for detailed investigation. Top two residues from Aβ_6_ in terms of binding frequency with Aβ_42_ are listed in parentheses.

**Figure 6:**
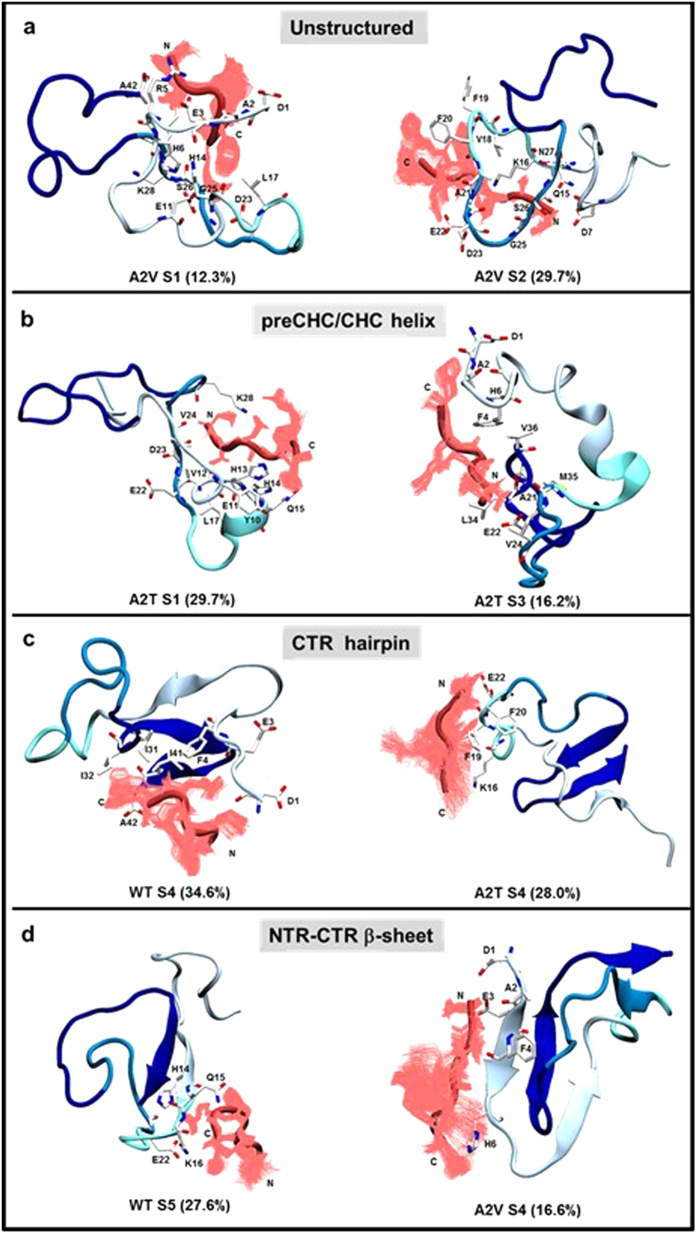
Alternative Aβ_42_ structures are increasingly populated due to hexapeptide binding. (**a**) Highly unstructured (within A2V-bound S1 and S2). (**b**) preCHC/CHC helix (within A2T-bound S1 and S3). (**c**) CTR-hairpin (within WT-bound and A2T-bound S4). (**d**) NTR-CTR β-sheet (within WT-bound S5 and A2V-bound S4). An RMSD-based clustering with 0.3 nm pairwise C_α_-RMSD cut-off was performed for each sub-population. Representative structures from the largest clusters are displayed. Relative population (in %) of the corresponding cluster within each state is reported in parenthesis. Peptides are displayed using cartoon representation. The Aβ_42_ peptide is colored according to the color scheme shown in Fig. 1a, while the Aβ_6_ peptide is colored in red. Different orientations of Aβ_6_ within each cluster are shown. Aβ_42_ residues that are within 0.4 nm (heavy atoms only) of the hexapeptide are displayed in licorice representation.

Concurrently, alternative conformations emerge on the hexapeptide-bound Aβ_42_ monomeric landscape, which substantially depend on the Aβ_6_ sequence (Fig. 5). Those alternative structures were further investigated in detail by performing a RMSD-based clustering on the S1–S5 sub-populations. Regions representing at least 10% of total population were only considered (Table 1). Representative structures are shown in Supplementary Fig. S10. Visual inspection of the representative structures, as extracted from clustering, revealed emergence of four main structural features within the alternative Aβ_42_ monomeric conformations: (i) an overall unstructured population visited mainly by the A2V-bound Aβ_42_, (ii) a preCHC/CHC helix populated in WT and A2T-bound monomer, (iii) a CTR β-hairpin, found in WT and A2T-bound monomer, and (iv) NTR-CTR β-sheet seen within WT and A2V-bound Aβ_42_ (see Fig. 6 and Supplementary Fig. S7). In the following, we discuss these four different classes of alternative structures in detail. Secondary structure and tertiary interaction profiles for those alternative populations are illustrated in Supplementary Fig. S8. A small, but non-negligible, 11% population consisting of a non-native register-shifted β-hairpin is seen in A2V-bound S5 structures, which is further discussed in the Supplementary Information (see Supplementary Fig. S9).

#### Disordered structures

The A2V-bound S1 and S2 states are consistent with an overall disordered structure (Fig. 6a and Supplementary Fig. S7), and together account for about 28% of the total population. Those Aβ_42_ structures mainly sample turn or coil conformation. The hexapeptide extensively engages with the CHC, 22–29 turn, or CTR residues, which directly competes with the CHC-CTR hairpin interactions needed to nucleate Aβ aggregation. V2 from the short peptide is frequently involved in these interactions.

#### Central helix structures

The preCHC/CHC helix is primarily seen in the A2T-bound S1, A2T-bound S3, and in WT-bound S2 states (Fig. 6b and Supplementary Fig. S7). This helical feature is most dominant in A2T-bound Aβ_42_, accounting for about 34% of the total ensemble (Table 1). Additional features, such as β-strand at the Aβ_42_ NTR or within the short peptide, are also seen (see Supplementary Fig. S7). In these structures, the hexapeptide mainly binds to the NTR, preCHC and the turn (residues 22–29) regions of Aβ_42_. H6 from the hexapeptide dominates the quaternary binding interface (Table 1) and contacts with NTR or preCHC residues.

#### CTR hairpin structures

The CTR-hairpin is the main characteristic of the WT-bound and A2T-bound S4 states (Fig. 6c and Supplementary Fig. S7), representing 18% and 20% of the total ensemble, respectively. The hairpin involves residues 30–41, with residues 34–38 generally in a turn conformation. It must be noted that the C-terminal hairpin with a G37/38 hinge has been reported in earlier simulation studies of free wild-type Aβ_42_^51, 56^. In some of those structures, NTR Aβ_42_ residues directly interact with the CTR hairpin, forming a β-sheet conformation. In some structures, the NTR, preCHC, and/or CHC residues adopt helical form. Within this sub-population, the hexapeptide directly contacts with the CTR hairpin or the CHC residues, again disrupting the CHC-CTR intramolecular interactions. F4 and R5 from Aβ_6_ dominate the intermolecular binding.

#### NTR-CTR β-sheet structures

The WT-bound S5 and the A2V-bound S4 states are characterized by an NTR-CTR β-sheet (Fig. 6d, Supplementary Fig. S7d). These sub-populations account for about 16.5% of the WT-bound and 23% of the A2V-bound ensemble (Table 1). The NTR of Aβ_42_ is mainly found to be in contact with the hexapeptide, whereas F4 from Aβ_6_ governs the quaternary association (Table 1).

## Discussion

In light of the AD-protective nature of familial A2V and A2T mutations in heterozygous carriers, therapeutic design based on the molecular basis of WT/A2V(A2T) Aβ cross-interaction appears promising. *In vivo* and *in vitro* studies have further confirmed the striking ability of the A2V and A2T Aβ variants to impede Aβ nucleation, neurotoxicity, and aggregation^37, 38, 41, 57, 58^ by directly interacting with WT Aβ. Remarkably, this inhibitory effect was found to be retained, even when WT Aβ_42_ was co-incubated with a short 1–6 WT or A2V Aβ variant, the effect being more prominent for the A2V variant^35, 45, 46^. Recently, short A2T NTR fragments of varying lengths have also been reported to inhibit fibrillization and rescue from toxicity^59^. A molecular characterization of the NTR hexapeptide binding with the full-length peptide is therefore crucial towards understanding of the aggregation-inhibitive and neuroprotective properties of those short peptides, which is useful for introducing new AD therapy. To our knowledge, the present simulation study reports for the first time the striking effect of the Aβ NTR hexapeptide binding on the WT Aβ_42_ monomeric landscape. Our results reveal remarkable differences between the WT, A2V, and A2T hexapeptide variant in terms of Aβ_42_ binding, and resulting structural changes.

While the overall disordered nature of Aβ_42_ persists, the transient CHC-CTR β-hairpin interactions that are implicated in Aβ aggregation and toxicity^18–20^ is strikingly reduced upon hexapeptide binding. Consequently, short-lived alternative populations emerge on the monomeric conformational landscape. The good agreement between the conformational landscape of the free and hexapeptide-bound monomer indicates involvement of conformational selection^60^ in the binding process. However, present simulations do not allow to quantify if, and to what extent, conformational selection governs binding. In the bound state, Aβ_6_ frequently populates a ‘fuzzy’ cloud of possible orientations around a transiently populated Aβ_42_ structure (Fig. 6 and Supplementary Fig. S7). In those fuzzy complexes^61^, the loss in overall binding enthalpy is somewhat compensated by a lower entropic loss. As a result of such dynamic interaction, long range electrostatic and transient physical contacts may increase^61^, leading to greater number of initial contacts and an increased capture radius^62^. Being an IDP, Aβ_42_ does not provide a unique binding groove to the hexapeptide, leading to promiscuous binding^63^. However, the Aβ_42_ NTR is often found engaged with the hexapeptide, which is desired, given the emerging pivotal role of NTR in Aβ structure, oligomerization/aggregation, associated toxicity, as well as interactions with anti-amyloid molecules^64–66^. A more flexible NTR is often associated with higher toxicity, as Aβ_42_ NTR is found more flexible than that of Aβ_40_ in monomer^67^ and dimer simulations^68^.

Four distinct structural features are consistently noticed within the emerging alternative Aβ_42_ populations due to hexapeptide binding: (i) an overall unstructured population, (ii) a preCHC/CHC helix, (iii) a CTR β-hairpin, and (iv) an NTR-CTR β-sheet. The relative propensity of hexapeptide-bound Aβ_42_ to visit these alternative sub-populations is strongly dependent on the Aβ_6_ sequence. For example, a central helix or a CTR β-hairpin is more frequently populated within the A2T-bound monomer, while A2V-bound Aβ_42_ is often disordered, or forms an intramolecular β-sheet involving both termini.

The emergence of a preCHC/CHC helix in the A2T or WT hexapeptide-bound Aβ_42_ (Fig. 6b and Supplementary Fig. S7) is of interest, since helix stabilization in this region has been reported to reduce fibril formation^69^ and counteract toxic oligomer formation^70^. Mutation such as V18A/F19A/F20A or addition of phospho-L-serine in the CHC region increases helical content, and subsequently reduces aggregation^69^. Therefore, enhanced helical Aβ_42_ population resulting from WT or A2T hexapeptide binding may impede Aβ_42_ aggregation, which is consistent with experimental findings^35, 45^.

A2V hexapeptide binding favors an overall unstructured population of monomeric Aβ_42_. Since Aβ monomer misfolding is implicated in formation of β-sheet rich oligomers^20^, the simulation results are in line with the experimental observation that binding of A2V hexapeptide inhibits β-structure formation in full-length Aβ^45^. Redirecting IDPs such as Aβ and α-synuclein towards stable, unstructured non-toxic oligomer formation has been associated with the aggregation and toxicity inhibiting effect of resveratrol^71^ and epigallocatechin gallate (EGCG) molecules^72^. Taken together, enhanced disorder in A2V Aβ_6_-bound Aβ_42_ might prevent toxic oligomer formation.

It is widely accepted that transiently formed β-strand/sheet-rich monomeric structures can promote aggregation and toxic oligomer formation in amyloidogenic proteins^73–77^, such as huntingtin exon1, α-synuclein and IAPP. Previous studies have also indicated a positive correlation between hydrophobic solvent exposure and aggregation propensity (and related toxicity)^14^. The hydrophobic solvent accessible surface area is consistently estimated to be smaller in the alternative Aβ_42_ structures with the C-terminal hairpin (2174 ± 62 Å^2^) or the NTR-CTR β-sheet (2375 ± 67 Å^2^), when compared to structures with a CHC-CTR β-hairpin (2567 ± 76 Å^2^). Thus, enhanced population of these atypical β-strand/sheet-rich conformations may help in lowering Aβ aggregation and toxicity. Commensurate to this speculation, a monomer with a C-terminal hairpin was more frequently seen in simulations of the protective A2T Aβ variant compared to the wild-type variant^42^. On the other hand, a double β-hairpin was often visited by the causative A2V Aβ monomer.

Redirecting Aβ monomers to atypical conformations appears a more feasible and attractive strategy for treating AD, compared to directly targeting Aβ production, as Aβ is essential in modulating synaptic activity, neuronal viability, and has shown potential antioxidative functions^78, 79^. The alternative conformations stabilized on the hexapeptide-bound Aβ landscape might be off the aggregation pathway, as indicated by the earlier experiments^69–72^. However, it has been proposed that the α-helical epitope, while present in a sub-population of monomeric Aβ, may be absent in oligomers and higher order aggregates. In fact, solution NMR in presence of helix promoting agents such as HFIP or SDS suggests helix formation only in Aβ monomers^80^. Thus, the A2T hexapeptide likely recognizes an epitope that is only present in a monomeric Aβ_42_ sub-population, resulting into a higher preference for monomer. On the other hand, the unstructured peptide conformation is found in a broad variety of Aβ species including oligomers and is related to lower toxicity^71, 72^. Therefore, targeting the unstructured epitope, as observed in the A2V hexapeptide binding, seems a more rational approach for AD therapeutic design. Our simulations reveal that the A2V short peptide binding stabilizes the unstructured monomer, subsequently sequestering the central and/or C-terminal hydrophobic regions and targeting the central turn region, thus eliminating the essential structural features needed for toxic Aβ oligomer and fibril formation. The unique population of disordered Aβ_42_, together with stronger binding affinity of the A2V hexapeptide, may explain its more pronounced effect on inhibiting β-sheet rich oligomer formation and aggregation of the full-length peptide, with respect to what was seen with the WT hexapeptide^45^.

A number of phenolic compounds, such as EGCG^72^, Congo Red dye^81^ and resveratrol^71^, have been previously identified to prevent Aβ aggregation and toxicity. Short peptide fragments derived from natural Aβ sequence have also been extensively studied as potential inhibitors of AD. The hydrophobic CHC derivatives^82^, while being effective fibrillation inhibitors, are reportedly less efficient in preventing oligomerization. On the other hand, CTR fragments^83, 84^ are more successful in mediating both oligomerization and fibrillogenesis through diverse mechanisms. Recent experiments establish the amyloid inhibition properties of the NTR Aβ fragment that is mainly hydrophilic in nature. The present study consistently illuminates the sequence-dependent, differential ability of the NTR hexapeptides to reconfigure the Aβ folding landscape away from disease-implicated structures. It will be intriguing to study the effect of length and sequence variation of the short peptide on the Aβ_42_ folding landscape, which will be addressed in future. In summary, our intriguing findings unravel the key structural features of the full-length Aβ monomer binding with N-terminal fragment derivatives, which offers novel molecular insights onto their amyloid inhibition properties and can further guide rational design of mechanism-based therapeutics for devastating protein aggregation diseases.

## Model and Methods

Description of the REMD protocol, including system setup, simulation parameters, etc. are provided in the Model and Methods section of the Supplementary Information. The Aβ_42_ monomer and the Aβ_6_ hexapeptide were placed together in a 5.7 × 5.7 × 5.7 nm^3^ sized cubic box containing ~5,600 water molecules, and charge-neutralized with Na^+^ and Cl^−^ atoms. The simulated system thus contains a total of about 17,400 atoms. A combination of the OPLS-AA force field^85^ and TIP3P water model^86^ was used for all simulations reported in this study, which has been reported to generate Aβ ensemble consistent with NMR measurements^47, 51^. During REMD, 64 replicas, each 200 ns long, spanning an exponentially distributed temperature range of 276.0–592.3 K were simulated in parallel and exchanged intermittently, resulting in an aggregate of 12.8 μs of conformational sampling for each system. Details of simulation equilibration and convergence assessment, structural characterization, clustering, binding energy calculations, PMF analysis, and error calculations are also reported in the Supplementary Information.

### Data Availability

All simulation data will be available upon request.

## Electronic supplementary material

Supplementary Information
