# Supplementary material for: Emergence of Alternative Structures in Amyloid Beta 1-42 Monomeric Landscape by N-terminal Hexapeptide Amyloid Inhibitors
Source: Sci Rep. 2017 Aug 30;7:9941. doi: 10.1038/s41598-017-10212-5 (PMC5577341; doi:10.1038/s41598-017-10212-5)
Supplement: Supplementary file 1 — Supplementary Information [file 41598_2017_10212_MOESM1_ESM.pdf]

Supplementary Information

Emergence of Alternative structures in Amyloid beta 1-42 Monomeric Landscape by N-terminal Hexapeptide Amyloid Inhibitors

Srirupa Chakraborty, Payel Das*

IBM Thomas J. Watson Research Center, Yorktown Heights, NY 10598

*Corresponding author email: daspa@us.ibm.com

Model and Methods

Molecular dynamics simulation at various resolutions[1-6] serves as a useful tool for the structural characterization of intrinsically disordered peptides, such as the Amyloid beta (Aβ). In this study we have performed extensive Replica Exchange Molecular Dynamics[7,8] (REMD) simulations to generate conformational ensemble of a single $A\beta_{42}$ monomer in presence of a WT, A2V, or A2T $A\beta_6$ variant. REMD is an enhanced sampling technique that has been successfully used to generate robust structural ensembles for intrinsically disordered proteins, such as the Aβ peptide, that lack a single native conformation and instead exists as dynamic ensemble of rapidly interconverting conformations[9-12]. In REMD, several identical replicas (*i.e.* copies) of the system are simulated in parallel over a wide range of temperature. Conformations populated at different temperatures are exchanged at frequent intervals following a Metropolis Monte Carlo criterion, so that the principle of detailed balance is satisfied. Employing this method ensures that the system does not get trapped in a local minimum on the free energy landscape[7].

The following protocol was used to set up the initial structure for the REMD simulation, which is similar to what was used in our earlier published simulations of the free $A\beta_{42}$ monomer variants[13]. A randomly collapsed $A\beta_{42}$ structure was generated from a short (~10 ps) MD simulation performed at 700 K in vacuum. The collapsed structure was then solvated, charge-neutralized by adding ions, energy minimized, and equilibrated for 1 ns in an NPT ensemble (300 K and 1 atm). The structure of the short WT $A\beta_6$ peptide was constructed using the first six residues of the full-length monomer as a template and ensuring that the phi and psi dihedral angles are consistent with a random coil structure. Next, the $A\beta_{42}$ monomer and the WT $A\beta_6$ hexapeptide were placed together in a 5.7 x 5.7 x 5.7 nm^3 sized cubic box that contains ~5,600 water molecules (Fig. 1b, main text), resulting in an effective peptide concentration of 10 mM. The minimum

distance (heavy atom only) between $A\beta_{42}$ and $A\beta_6$ in the initial structure was at least 1.5 nm. The peptide termini were charged, and four Na^+ ions were added to charge-neutralize the system. A similar protocol was employed to generate the initial solvated structure of the $A\beta_{42}$ in presence of the A2V or A2T hexapeptide variant, in which the sidechain of residue 2 of the hexapeptide was accordingly mutated *in silico* using the Mutate plugin of the VMD software[14]. Prior to the REMD run, all solvated systems were first subjected to a 10,000-step energy-minimization, followed by a 10 ns long equilibration run in an NPT ensemble (300 K and 1 atm). Finally, constant volume REMD simulations were run for at least 200 ns per replica. A total of 64 replicas within an exponentially distributed temperature range[15] of 276-592.3 K were used, and replica exchange attempts were made every 4 ps, resulting into an average exchange ratio of 19% that is constant over the temperature range.

The specific temperatures at which replicas were run are:

276.0, 278.7, 281.5, 284.3, 287.2, 290.1, 293.0, 296.0, 299.0, 302.1, 305.2, 308.4, 311.7, 314.9, 318.3, 321.7, 325.1, 328.6, 332.2, 335.8, 339.5, 343.3, 347.1, 351.0, 355.0, 359.0, 363.1, 367.3, 371.6, 375.9, 380.4, 384.9, 389.5, 394.2, 399.0, 403.8, 408.8, 413.9, 419.1, 424.4, 429.8, 435.3, 440.9, 446.6, 452.5, 458.5, 464.6, 470.8, 477.1, 483.6, 490.3, 497.1, 504.0, 511.1, 518.3, 525.8, 533.4, 541.1, 549.1, 557.3, 565.7, 574.3, 583.1, 592.3.

The system was coupled to a Nosé-Hoover heat bath to maintain constant temperature between the swaps. Periodic boundary conditions were applied and the particle-mesh Ewald (PME) method was used to treat long-range electrostatics[16]. A 1.0 nm cut-off distance was used for van der Waals interactions. The bonds were constrained using LINCS[17] and SETTLE[18] algorithms, and an integration time-step of 2 fs was used. A combination of OPLS-AA force field[19] and TIP3P water model[20] was used, which has been previously reported to generate

disordered protein structural ensembles, such as Aβ, in agreement with NMR experiments[21-23]. This combination is found to be well suited for simulating Aβ fragments[24] and full-length Aβ peptides[25], and has been also used in our previously published Aβ monomer and dimer simulations[13,26]. The simulations were performed using the GROMACS 4.5.4 software[27] in IBM BlueGene/Q supercomputers.

Conformational analysis: The secondary structure of the peptide was calculated using the STRIDE program[28]. The C_α-C_α tertiary contact between a pair of residues was defined by using a distance cut-off of 0.8 nm between the C_α atoms. Only non-sequential contacts ($|i-j| \geq 3$) were considered for tertiary interactions. A salt bridge was defined using a distance cut-off of 0.32 nm between the side-chain oxygen atom of an acidic residue and the side-chain nitrogen atom of a basic residue. An inter-peptide contact was defined by using a distance cut-off of 0.5 nm (heavy atoms only). The hexapeptide was considered to be bound to the $A\beta_{42}$ monomer in a structure, only if ≥ 25 intermolecular contacts were present. The $A\beta_{42}$ conformational ensemble was clustered using the Root Mean Squared Deviation (RMSD) as a similarity measure. The Daura algorithm[29] with a 0.45 nm C_α-RMSD cut-off between two conformations was used for clustering of the whole production ensemble (Fig. S2). All peptide structures were rendered using VMD software[14].

Solvent Accessible Surface Area: The hydrophobic solvent accessible surface area (SASA) was calculated using the VMD plugin with a probe radius of 1.4 Å[14]. The average SASA value for the CHC-CTR β-hairpin structure was calculated using the free $A\beta_{42}$ (structures corresponding to regions 6 and 7 of Fig. S6). The average SASA value for CTR-hairpin structures was obtained from the largest cluster of WT-bound and A2T-bound S4 sub-populations. For the NTR-CTR β-

sheet structures, the largest cluster of WT-bound S5 and A2V-bound S4 sub-populations were used.

Binding energy calculation: The average binding free energies (ΔG_{bind}) between $A\beta_{42}$ and $A\beta_6$ were calculated using the MM-PBSA method[30,31]. In this technique, the total binding energy is a cumulative of the molecular-mechanics energy (van der Waals and electrostatics) and solvation (non-polar and polar solvation) energy terms. The MM-PBSA method does not account for the entropic contribution to binding. The non-polar solvation energy was estimated using a model based on a combination of solvent-accessible surface area and solvent accessible volume calculation[30,32]. The molecular dielectric surface was defined by a probe radius of 1.4 Å. The dielectric constants of the protein interior and the aqueous environment were set to 2 and 80, respectively, and the ionic concentration set to 150 mM. Standard deviations (SD) were estimated by using a bootstrap approach, and standard errors of mean (SEM) were computed from the SD derived from an ensemble of ~30,000 structures.

PMF analysis and clustering: Potential of mean force (PMF, W(X)) was defined by the equation $W(x) = -RT.\log(p(X))$, where *X* is a set of reaction coordinates and *p(X)* is the probability. Following reaction coordinates were defined for this purpose: (i) the total number of residues from the CHC and CTR in an extended β-strand conformation ($N\beta_{CHC+CTR}$); and (ii) the number of C_α contacts between CHC and CTR ($NC_{CHC-CTR}$); both normalized to unity. Six sub-populations (S1 through S6) were defined on the PMF landscape that correspond to the regions having PMF energy < -4 kcal/mol (see Fig. 5, main text). The arbitrary boundaries used to define these sub-populations are reported in Table S3. The sub-populations that individually represent ≥10% of total production ensemble were further analyzed. An RMSD-based clustering was performed using the Daura algorithm[29] and a 0.3 nm pairwise C_α-RMSD cut-off on each of those sub-populations.

Error analysis: Standard errors of mean (SEM) were estimated for secondary, tertiary, and quaternary structural features, as well as for the relative population of S1-S6 states on the PMF landscape. These SEM values were obtained from standard deviations estimated by dividing the simulation data into two (or four) 35 ns long, non-overlapping blocks between 60 – 130 (or 60-200) ns. The SEM in the probability difference plots (secondary and tertiary structure analyses, Figs. 2 and 3, main text) were estimated by following the standard rule of error propagation.

Equilibration and convergence assessment of REMD ensemble

Along with the Root Mean Square Deviation (RMSD) analysis described in the main text (Fig. 1c), we performed the following evaluations to further validate the REMD ensemble. To ensure that the choice of initial peptide structure did not bias the final results, we checked the evolution of intermolecular distance between the centers of mass (COM) of the full-length peptide and the hexapeptide. The evolution of intermolecular distance for the 308.4 K replica is shown in Fig. S1. The interpeptide distance equilibrates to about 1.2 nm by 50 ns, and fluctuates steadily around this mean with a standard deviation of ± 0.4 nm. This observation suggests that the ensemble populated after 50 ns is independent of the initial conformation. Therefore, we considered the 60-200 ns portion of REMD trajectories for further analysis.

Next, we compared the probability distributions of (i) the radius of gyration (R_g); (ii) the number of intra-peptide CHC-CTR C_α contacts ($N_{CHC\text{-}CTR}$); and (iii) the residue-wise turn propensity of $A\beta_{42}$ over two time intervals, 60 – 130 ns and 60 – 200 ns, in order to ensure that the simulations have reasonably converged. Results for the 308.4 K replica are shown in Fig. S1 and Table S1. For all three distributions, the overall range and the major features remain constant

between the two time-intervals. The mean R_g value is 1.04 nm, 1.11 nm, and 1.05 nm for the WT-bound, A2V-bound, and A2T-bound $A\beta_{42}$ and remain unchanged over the two time-windows of 60–130 ns and 60–200 ns. The average number of CHC-CTR contacts is about 12 for all three hexapeptide-bound systems. The turn propensity distributions are virtually indistinguishable between the different time windows, suggesting good convergence of the REMD trajectories.

Size and disorder of the production ensemble

The R_g distribution of the production ensemble is distributed around the mean value of 1.06 ± 0.12 nm for free, 1.05 ± 0.07 nm for WT-bound, 1.07 ± 0.10 nm for A2V-bound, and 1.05 ± 0.08 nm for A2T-bound $A\beta_{42}$ (Fig. S2). This value matches well with earlier experimental[33] and simulations studies[1,21,22] and is consistent with a collapsed globule. Interaction with WT or A2T hexapeptides increases the population around the mean R_g value, while the distribution corresponding to the A2V-bound peptide is similar to that of the free $A\beta_{42}$ monomer. Since $A\beta_{42}$ is an IDP, we performed a RMSD-based clustering for the $A\beta_{42}$ ensembles to compare the structural disorder (see Methods and Fig. S2). It is interesting to observe that the population distribution of the largest 50 clusters is similar for all four $A\beta_{42}$ systems, indicating that the overall structural heterogeneity is not strongly affected by the NTR hexapeptide binding.

Register-shifted β-hairpin within A2V S5 structures

Analysis of A2V-bound S5 structures that present 11.6% of the total ensemble reveals a β-strand rich population (Fig. S9). The β-hairpin is comprised of β-strands involving residues 18-22

and 27-31 and a short turn region around residues 23-26. This structure is different from the transient CHC-CTR hairpin often observed within free Aβ_{42} S6 structures (Fig. S6), as the hairpin within A2V-bound S5 Aβ_{42} shows a register shift towards C-terminus and a shorter turn. The hexapeptide mainly interacts through residues D1 and V2 with NTR, CHC or CTR residues of the Aβ_{42} monomer.

Representative structure of S1-S6 sub-population

The most representative structure of each significant (>10%) sub-population of free and WT/A2V/A2T bound Aβ_{42} is shown in Figure S10. While the free and the A2V-bound peptides in S1 are overall unstructured, the A2T-bound S1 structure has the preCHC/CHC helix. The WT-bound S2 structure also acquires the preCHC/CHC helix conformation, while the A2V-bound S2 remains unstructured. Only the A2T-bound peptide shows a significant population of the S3 region and frequently visits the preCHC/CHC helix conformation. The free Aβ_{42} structure from the S4 region forms a β-hairpin with residues 23-40 with a short helical turn in the CHC region. While the WT-bound and A2T-bound S4 structures are comprised of the CTR-hairpin, A2V-bound S4 structure forms the NTR-CTR β-sheet. Within S5 sub-population, WT-bound structure also forms the NTR-CTR β-sheet, while A2V-bound structure acquires the register-shifted β-hairpin conformation. The S6 Aβ_{42} structure (bound or free) consistently exhibit the disease-implicated CHC-CTR hairpin conformation. Taken together, our analysis confirms the sequence-dependent effect of the hexapeptide interaction on the Aβ_{42} structure.

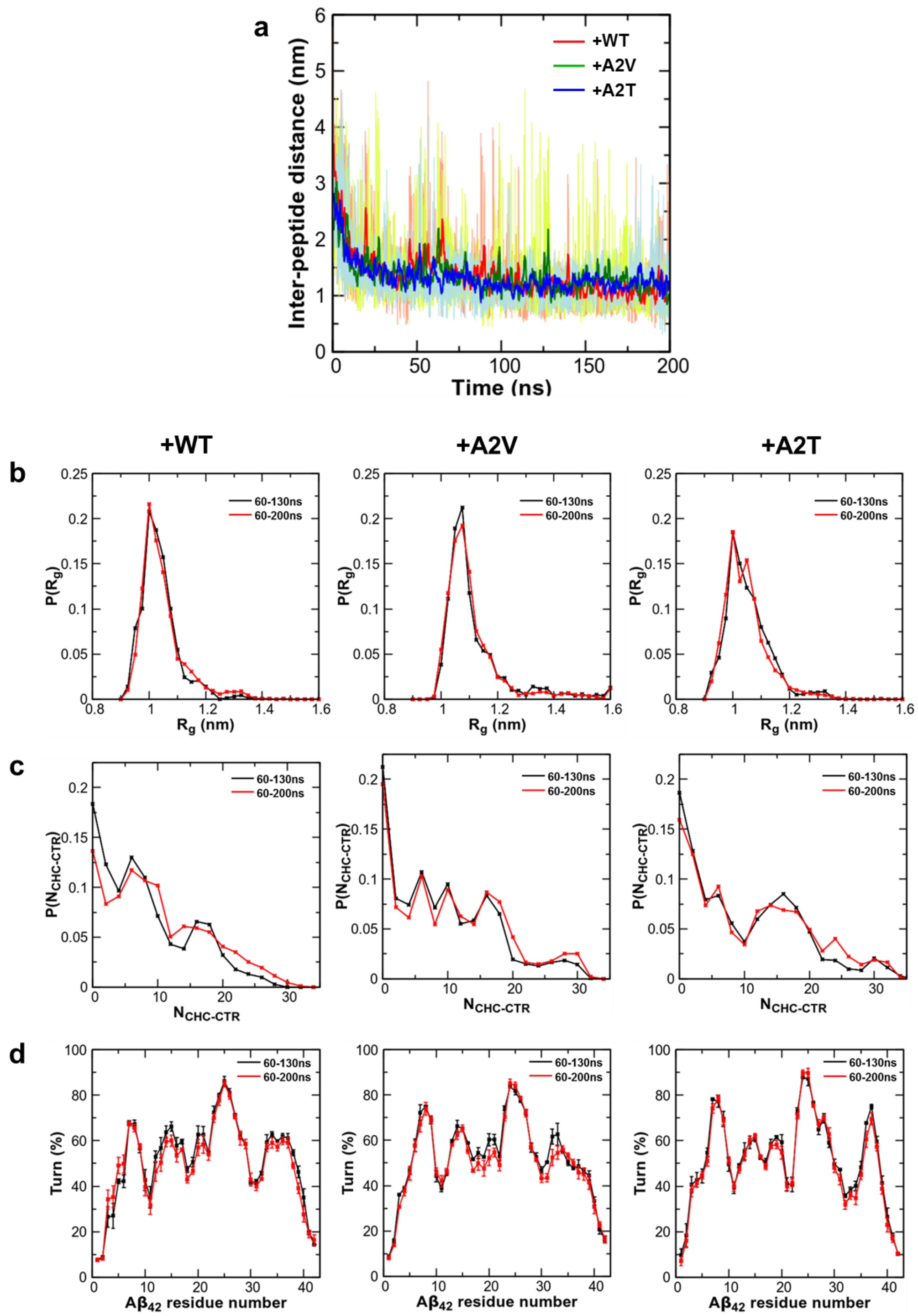

Figure S1:

REMD equilibration and convergence analysis. Results are shown for the replica corresponding to 308.4 K. Moving averages of 1 ns are shown. (a) Intermolecular distance (in nm) between the centers of mass (COM) of $A\beta_{42}$ and $A\beta_6$ as a function of simulation time (in ns). The raw data (WT-bound: light brown,

A2V-bound: light green, A2T-bound: light blue) are also plotted. (b) Probability distribution of radius of gyration, R_g (in nm) of $A\beta_{42}$. (c) Probability distribution of number of intra-peptide CHC-CTR C_α contacts, $N_{CHC\text{-}CTR}$, within $A\beta_{42}$. (d) Residue-wise turn propensity (in %) of $A\beta_{42}$. Standard errors of mean were estimated by dividing the data into two or four 35 ns long blocks (see Methods). Results for 60-130 ns are shown in black and those for 60-200 ns are shown in red. Results corresponding to WT-bound, A2V-bound, and A2T-bound $A\beta_{42}$ are shown in left, center and right panel, respectively.

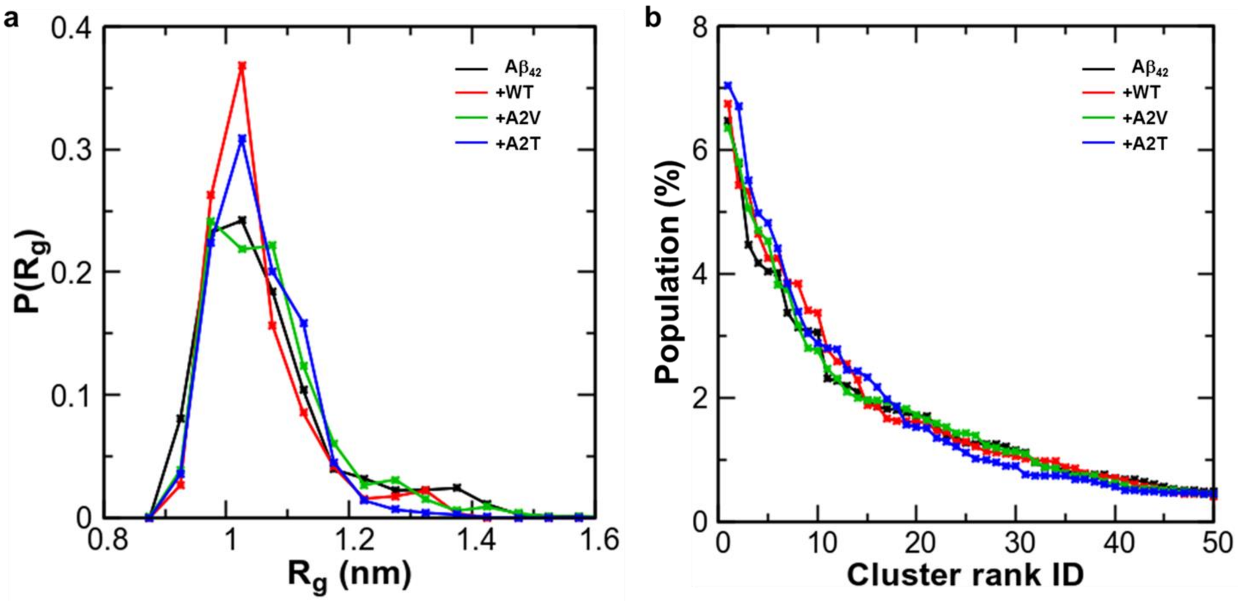

Figure S2:

(a) Probability distribution of radius of gyration, R_g, of $A\beta_{42}$, as estimated from the production ensemble, and (b) Population distribution of largest 50 clusters of $A\beta_{42}$ structures, as obtained using a RMSD-based clustering of the production ensemble using a pairwise C_α RMSD cut-off of 0.45 nm.

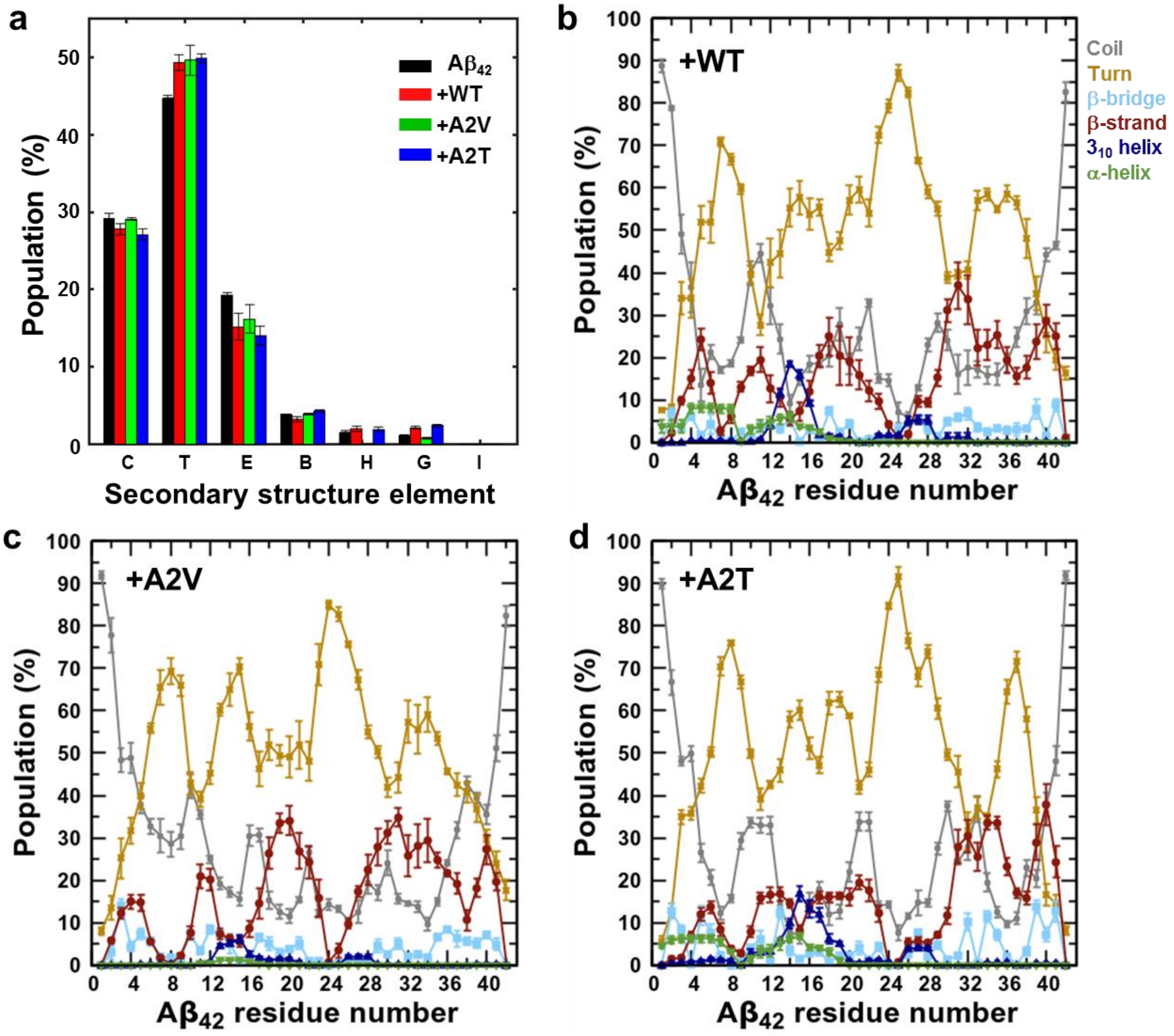

Figure S3:

Secondary structure analysis of Aβ_{42}. (a) Overall population (in %) of secondary structural elements (C = coil, T = turn, E = β-strand, B = β-bridge, H = α-helix, G = 3_{10}-helix, I = π-helix). (b-d) Residue-wise population distribution (in %) of secondary structure elements in (b) WT-bound, (c) A2V-bound, and (d) A2T-bound Aβ_{42}.

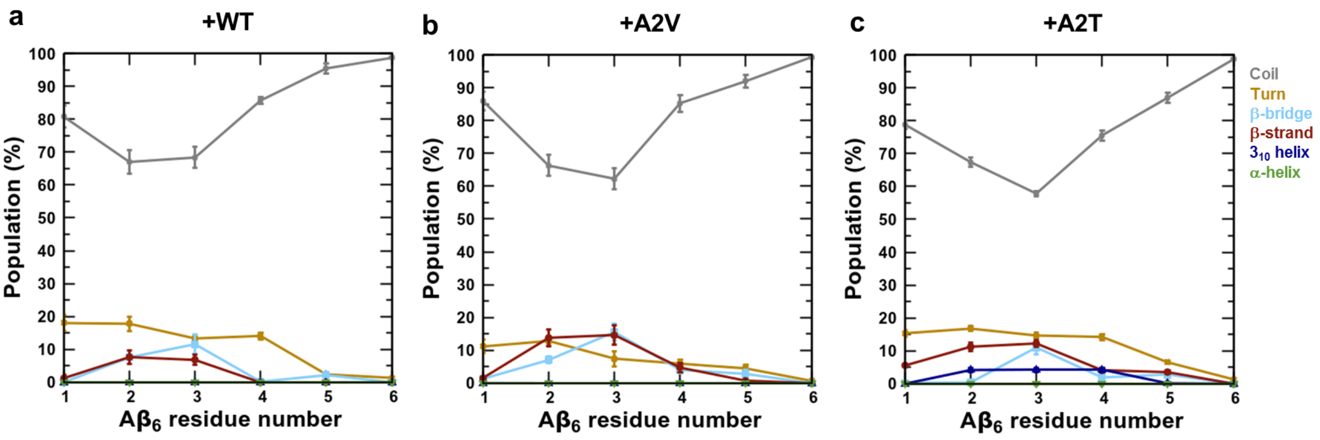

Figure S4:

Secondary structure analysis of Aβ$_6$. (a-c) Residue-wise population distribution (in %) of secondary structure elements for the (a) WT, (b) A2V, and (c) A2T hexapeptide.

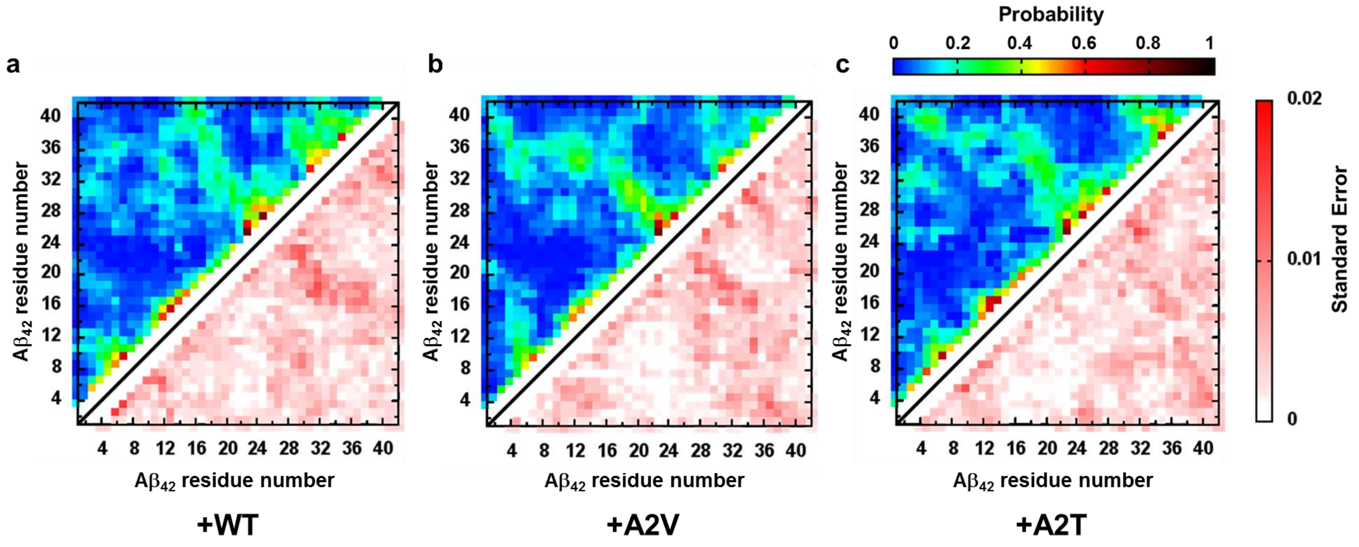

Figure S5:

Tertiary contact map of hexapeptide-bound Aβ$_{42}$ monomer. (a-c) Ensemble-averaged intramolecular C$_\alpha$ contact map (upper triangles) for (a) WT-bound, (b) A2V-bound, and (c) A2T-bound Aβ$_{42}$. Only non-sequential contacts, i.e., $|i - j| \geq 3$, are shown. Associated standard error values (see Methods) are displayed in the respective lower triangles.

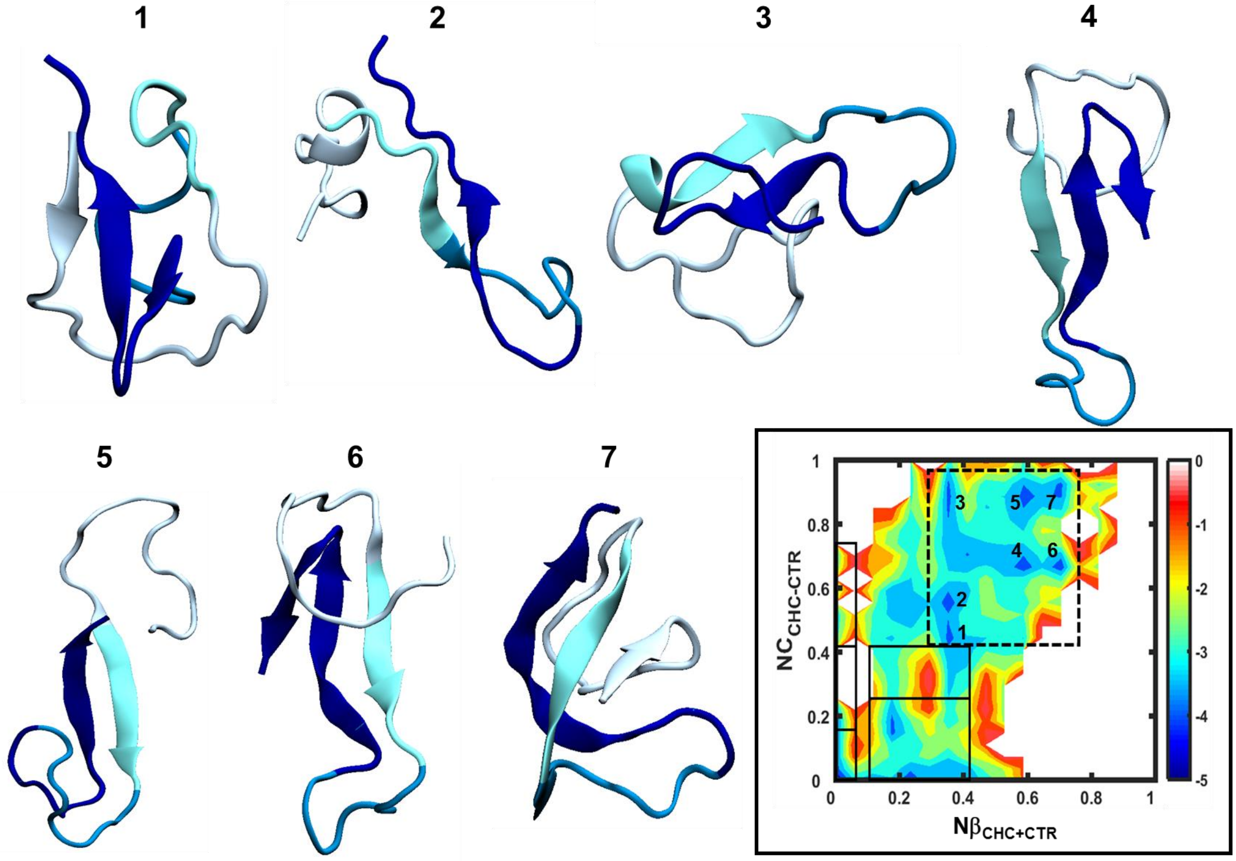

Figure S6:

Structural diversity within the S6 sub-population of free Aβ_{42} monomer. The PMF plot of free Aβ_{42} is shown at bottom right. The seven structures correspond to seven regions (marked 1 to 7) with PMF value < -4 kcal/mol. Most representative structure of the largest cluster is shown.

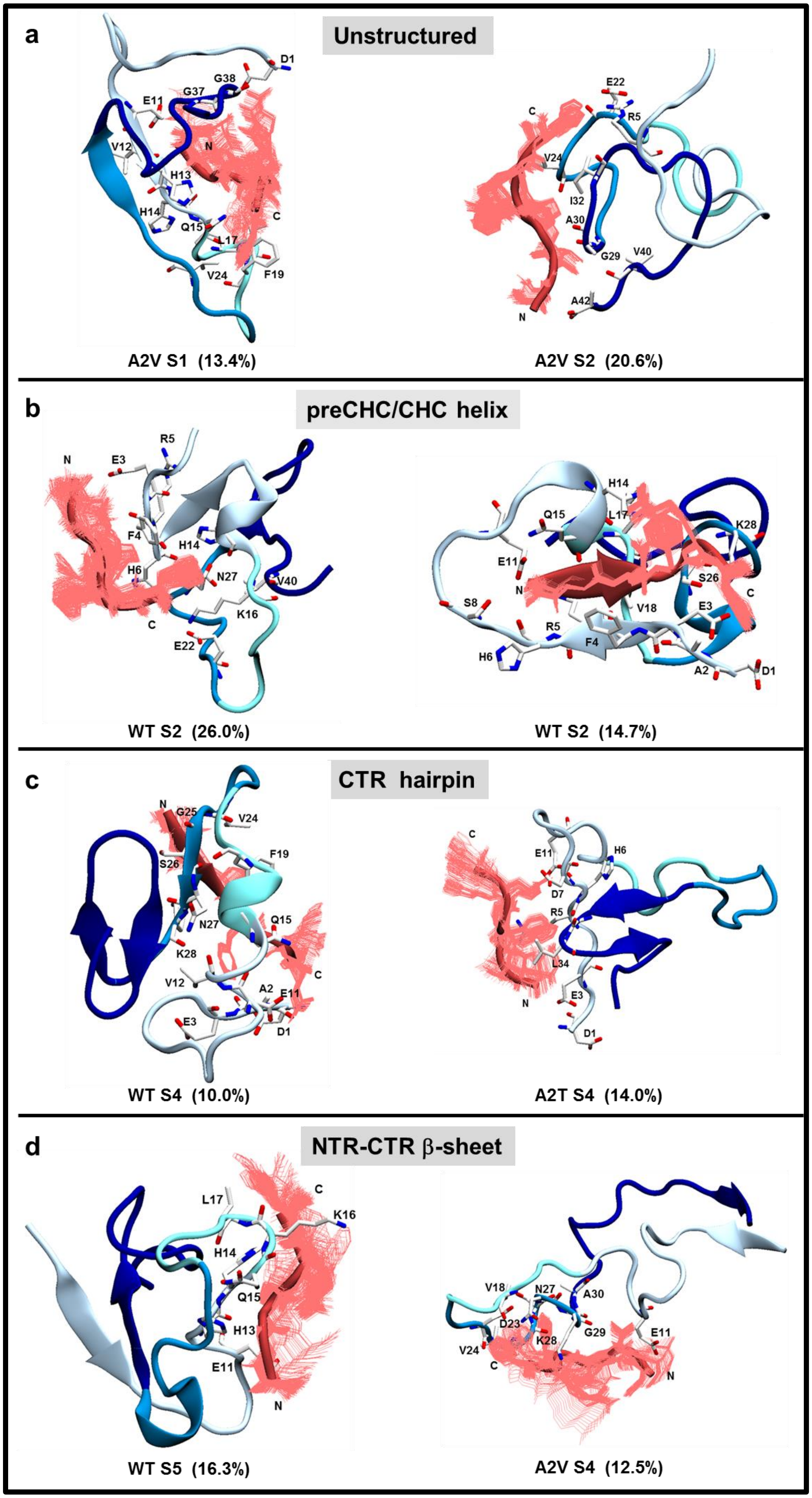
a
Unstructured
D1
G38
G37
E11
V12
N
H13
H14
Q15
C
L17
V24
F19
A2V S1 (13.4%)
E22
C
R5
V24
I32
A30
V40
G29
A42
N
A2V S2 (20.6%)
b
preCHC/CHC helix
R5
N
E3
F4
H14
H6
N27
V40
K16
C
E22
WT S2 (26.0%)
H14
Q15
L17
K28
E11
N
S26
C
S8
V18
E3
R5
F4
H6
A2
D1
WT S2 (14.7%)
c
CTR hairpin
N
G25
V24
F19
S26
N27
Q15
K28
C
V12
A2
E11
E3
D1
WT S4 (10.0%)
C
E11
H6
D7
R5
L34
N
E3
D1
A2T S4 (14.0%)
d
NTR-CTR β-sheet
L17
C
K16
H14
Q15
H13
E11
N
WT S5 (16.3%)
V18
N27
A30
D23
K28
G29
E11
V24
C
N
A2V S4 (12.5%)

Figure S7:

Additional snapshots of alternative Aβ_{42} structures emerging due to hexapeptide binding. (a) Overall unstructured conformations (A2V-bound S1 and S2 states). (b) preCHC/CHC helix conformations (WT-bound S2 state). (b) CTR-hairpin conformations (WT-bound and A2T-bound S4). (c) NTR-CTR β-sheet conformations (WT-bound S5 and A2V-bound S4). An RMSD-based clustering with 0.3 nm pairwise C_{α}-RMSD cut-off was performed for each sub-population. Representative structures from largest clusters are displayed. Relative population (in %) of the corresponding cluster within each state is reported in parenthesis. Peptides are displayed using cartoon representation. The Aβ_{42} peptide is colored according to the color scheme shown in Fig. 1a, while the Aβ_{6} peptide is colored in red. Different orientations of Aβ_{6} within each cluster are shown. Aβ_{42} residues that are within 0.4 nm (heavy atoms only) of the hexapeptide are displayed in licorice representation.

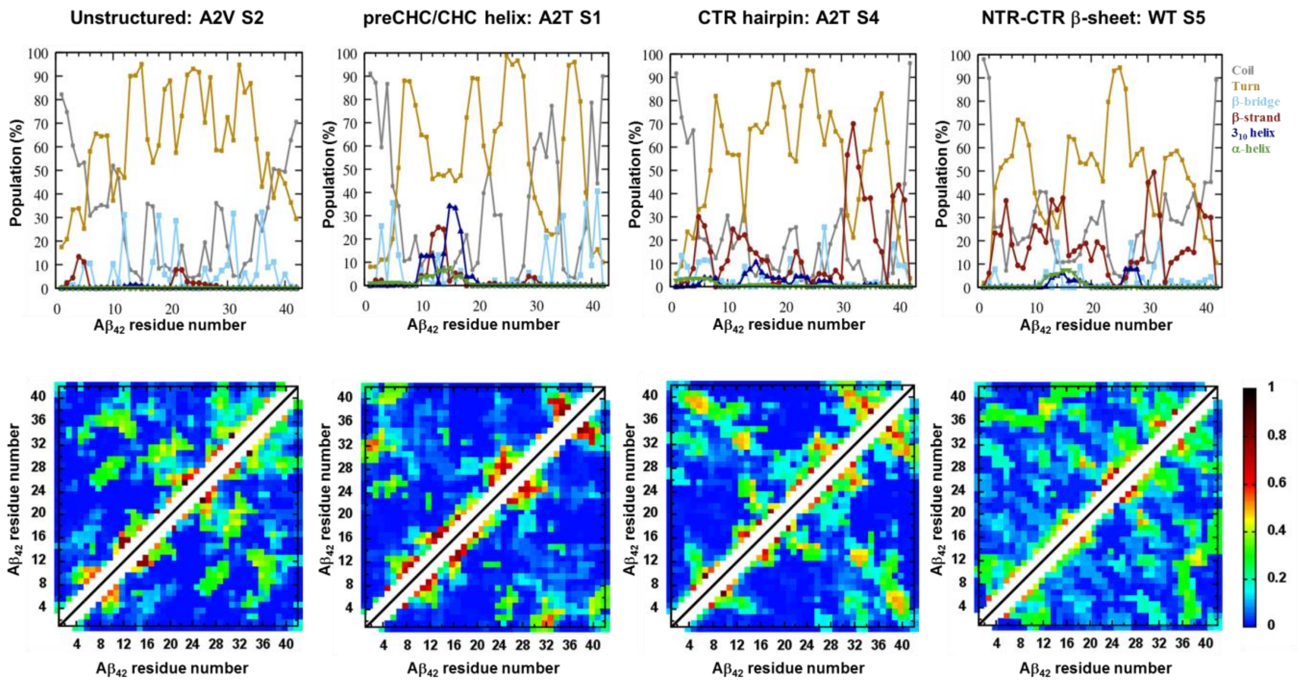

Figure S8:

Residue-wise secondary structure profile, and ensemble-averaged intramolecular C_α contact map of the hexapeptide-bound $A\beta_{42}$ monomer with atypical structural features.

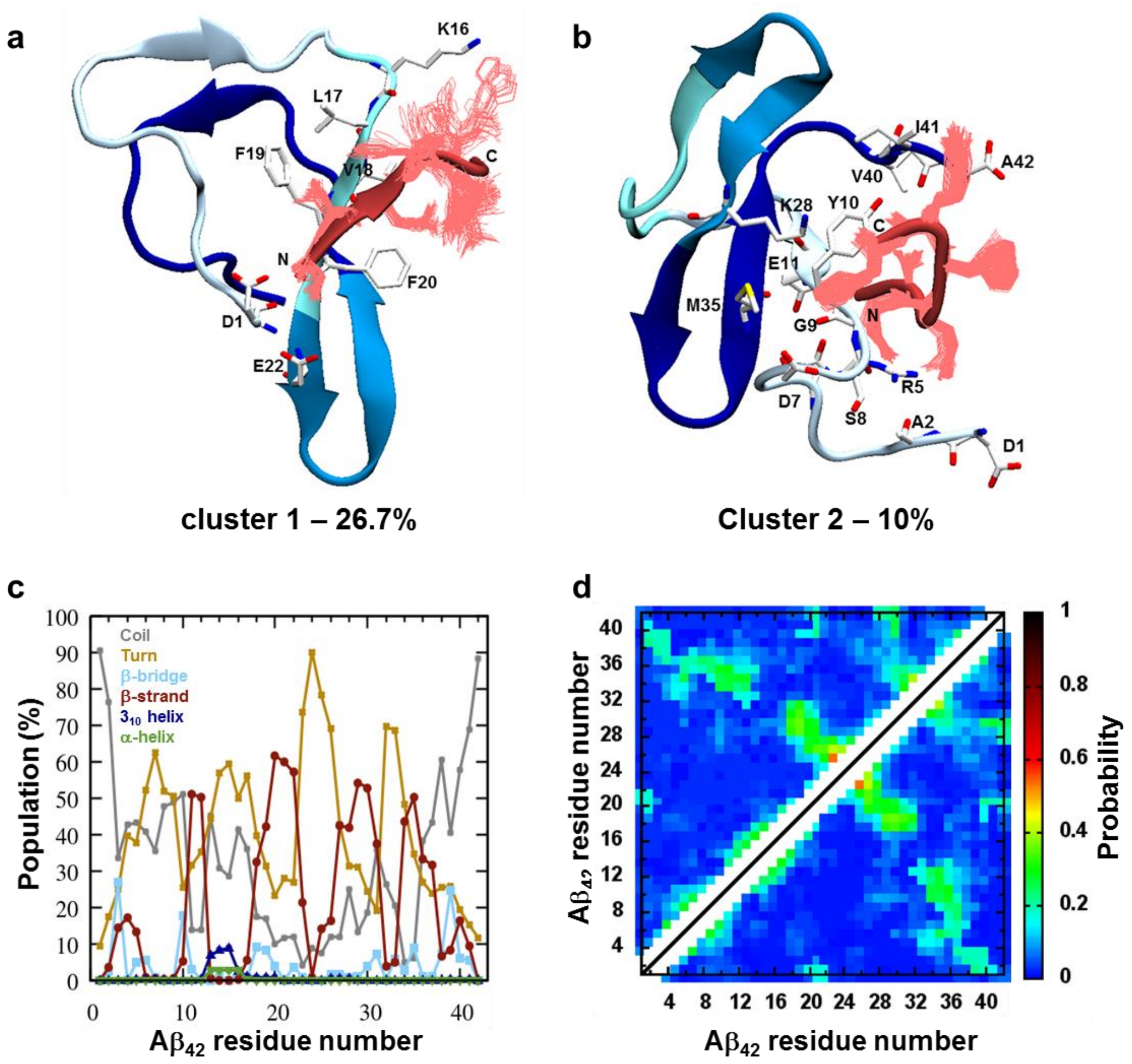

Figure S9: Structural Characterization of +A2V S5 sub-population. (a, b) Representative conformation of the two largest clusters (with 26.7 % and 10% population), as extracted using a RMSD-based clustering. (c) Residue-wise secondary structure profile and (d) ensemble-averaged intramolecular Cα contact map of Aβ_{42}.

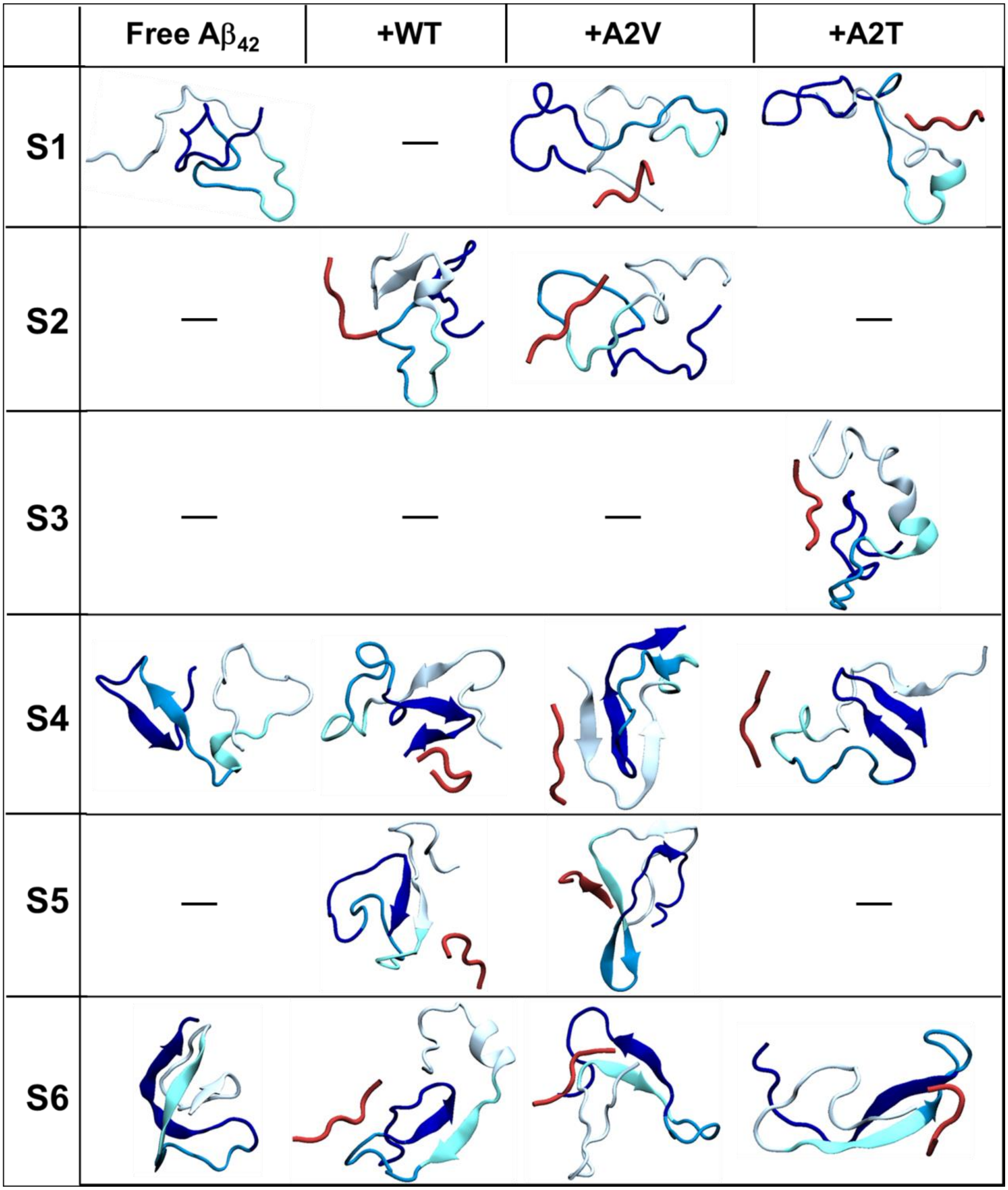

Figure S10: Representative structures from each sub-population S1 – S6, that is populated above 10%. The color code is the same as that used in Fig. S7.

	+WT		+A2V		+A2T	
	60-130 ns	60-200 ns	60-130 ns	60-200 ns	60-130 ns	60-200 ns
R_g (nm)	1.04 ± 0.07	1.04 ± 0.08	1.12 ± 0.13	1.11 ± 0.11	1.05 ± 0.08	1.05 ± 0.07
$N_{CHC\text{-}CTR}$	13 ± 4	12 ± 4	13 ± 5	12.5 ± 5	12 ± 3	12 ± 3
Turn (%)	51.8 ± 2.8	50.1 ± 2.6	52.7 ± 2.6	50.7 ± 2.6	52.1 ± 2.9	50.7 ± 3.0

Table S1: REMD convergence assessment, as estimated from results (mean ± standard deviation) obtained over 60-130 ns and 60-200 ns time-window.

	+WT	+A2V	+A2T
Van der Waals Energy	-26.6 ± 0.1	-27.9 ± 0.1	-27.5 ± 0.1
Electrostatic Energy	-25.0 ± 0.2	-36.8 ± 0.2	-42.6 ± 0.2
Polar Solvation Energy	73.8 ± 0.2	82.6 ± 0.3	87.4 ± 0.2
SASA Energy	-4.2 ± 0.0	-4.5 ± 0.0	-4.4 ± 0.0
SAV Energy	-36.7 ± 0.1	-39.9 ± 0.1	-39.2 ± 0.1
Binding Energy	-18.7 ± 0.1	-26.5 ± 0.1	-26.3 ± 0.1

Table S2: The total interpeptide binding free energy, as well as contribution of individual energy component, as estimated using the MM-PBSA method (in kcal/mol; ± standard error of mean). ΔE_{VdW} = van der Waals; ΔE_{elec} = electrostatic; ΔE_{solv} = polar solvent; ΔE_{SASA} = solvent-accessible surface area; ΔE_{SAV} = solvent-accessible volume terms. ΔG_{bind} = total binding free energy.

	S1	S2	S3	S4	S5	S6
$N\beta_{CHC+CTR}$ range	0.00 – 0.06	0.00 – 0.06	0.00 – 0.06	0.12 – 0.42	0.12 – 0.42	0.29 – 0.77
$NC_{CHC\text{-}CTR}$ range	0.00 – 0.15	0.15 – 0.41	0.41 – 0.74	0.00 – 0.25	0.25 – 0.41	0.41 – 0.96

Table S3: Boundaries of S1-S6 sub-populations on the PMF landscape.

References

1 Baumketner, A. *et al.* Amyloid β-protein monomer structure: A computational and experimental study. *Protein Science* **15**, 420-428 (2006).

2 Urbanc, B. *et al.* In silico study of amyloid beta-protein folding and oligomerization. *Proc Natl Acad Sci USA* **101**, 17345-17350 (2004).

3 Krone, M. G. *et al.* Effects of familial Alzheimer's disease mutations on the folding nucleation of the amyloid beta-protein. *Journal of molecular biology* **381**, 221-228 (2008).

4 Ball, K. A. *et al.* Homogeneous and heterogeneous tertiary structure ensembles of amyloid-beta peptides. *Biochemistry* **50**, 7612-7628 (2011).

5 Cote, S., Derreumaux, P. & Mousseau, N. Distinct morphologies for amyloid beta protein monomer: Abeta1-40, Abeta1-42, and Abeta1-40 (D23N). *Journal of Chemical Theory and Computation* **7**, 2584-2592.

6 Dorosh, L. & Stepanova, M. Probing oligomerization of amyloid beta peptide in silico. *Mol Biosyst* **13**, 165-182 (2016).

7 Sugita, Y. & Okamoto, Y. Replica-exchange molecular dynamics method for protein folding. *Chemical Physics Letters* **314**, 141-151 (1999).

8 Chodera, J. D. & Shirts, M. R. Replica exchange and expanded ensemble simulations as Gibbs sampling: simple improvements for enhanced mixing. *J Chem Phys* **135**, 194110 (2011).

9 Miyashita, N., Straub, J. E. & Thirumalai, D. Structures of beta-Amyloid Peptide 1-40, 1-42, and 1-55-the 672-726 Fragment of APP-in a Membrane Environment with Implications for Interactions with gamma-Secretase. *Journal of the American Chemical Society* **131**, 17843-17852 (2009).

10 Qiao, Q., Bowman, G. R. & Huang, X. Dynamics of an Intrinsically Disordered Protein Reveal Metastable Conformations That Potentially Seed Aggregation. *Journal of the American Chemical Society* **135**, 16092-16101, doi:10.1021/ja403147m (2013).

11 Sgourakis, N. G. *et al.* Atomic-level characterization of the ensemble of the Aβ(1-42) monomer in water using unbiased Molecular Dynamics simulations and spectral algorithms. *J. Mol. Biol.* **405**, 570-583 (2011).

12 Fukunishi, H., Watanabe, O. & Takada, S. On the Hamiltonian replica exchange method for efficient sampling of biomolecular systems: Application to protein structure prediction. *J. Chem. Phys.* **116**, 9058-9067 (2002).
13 Das, P., Murray, B. & Belfort, G. Alzheimer's protective A2T mutation changes the conformational landscape of the Abeta1-42 monomer differently than does the A2V mutation. *Biophys J* **108**, 738-747 (2015).
14 Humphrey, W., Dalke, A. & Schulten, K. VMD: visual molecular dynamics. *Journal of molecular graphics* **14**, 33-38 (1996).
15 Patriksson, A. & van der Spoel, D. A temperature predictor for parallel tempering simulations. *Physical Chemistry Chemical Physics* **10**, 2073-2077 (2008).
16 Deserno, M. & Holm, C. How to mesh up Ewald sums. II. An accurate error estimate for the particle-particle-particle-mesh algorithm. *J Chem Phys* **109**, 7694-7701 (1998).
17 Hess, B., Bekker, H., Berendsen, H. J. C. & Fraaije, J. G. E. M. LINCS: A linear constraint solver for molecular simulations. *Journal of Computational Chemistry* **18**, 1463-1472 (1997).
18 Miyamoto, S. & Kollman, P. A. Settle: An analytical version of the SHAKE and RATTLE algorithm for rigid water models. *Journal of Computational Chemistry* **13**, 952-962 (1992).
19 Jorgensen, W. L., Maxwell, D. & Tirado-Rives, J. Development and testing of the OPLS all-atom force field on confromational energetics and properties of organic liquids. *J. Am. Chem. Soc.* **118**, 11225-11236 (1996).
20 Jorgensen, W. L., Chandrasekhar, J., Madura, J. D., Impey, R. W. & Klein, M. L. Comparison of simple potential functions for simulating liquid water. *J Chem Phys* **79**, 926-935 (1983).
21 Rosenman, D. J., Connors, C. R., Chen, W., Wang, C. & Garcia, A. E. Abeta Monomers Transiently Sample Oligomer and Fibril-Like Configurations: Ensemble Characterization Using a Combined MD/NMR Approach. *J Mol Biol* **425**, 3338-3359 (2013).
22 Carballo-Pacheco, M. & Strodel, B. Comparison of force fields for Alzheimer's A beta42: A case study for intrinsically disordered proteins. *Protein Sci* **26**, 174-185 (2017).
23 Hoffmann, K. Q., McGovern, M., Chiu, C. C. & de Pablo, J. J. Secondary Structure of Rat and Human Amylin across Force Fields. *PLoS One* **10**, e0134091 (2015).
24 Nguyen, P. H., Li, M. S. & Derreumaux, P. Effects of all-atom force fields on amyloid oligomerization: replica exchange molecular dynamics simulations of the A[small beta]16-22 dimer and trimer. *Physical Chemistry Chemical Physics* **13**, 9778-9788 (2011).
25 Tarus, B. *et al.* Structures of the Alzheimer’s Wild-Type Aβ1-40 Dimer from Atomistic Simulations. *The Journal of Physical Chemistry B* **119**, 10478-10487 (2015).
26 Das, P., Chacko, A. R. & Belfort, G. Alzheimer’s Protective Cross-Interaction between Wild-Type and A2T Variants Alters Aβ42 Dimer Structure. *ACS Chemical Neuroscience* (2016).
27 Hess, B., Kutzner, C., Van Der Spoel, D. & Lindahl, E. GROMACS 4: Algorithms for highly efficient, load-balanced, and scalable molecular simulation. *Journal of Chemical Theory and Computation* **4**, 435-447 (2008).
28 Frishman, D. & Argos, P. Knowledge-based protein secondary structure assignment. *Proteins* **23**, 566-579 (1995).
29 Daura, X. *et al.* Peptide Folding: When Simulation Meets Experiment. *Angewandte Chemie International Edition* **38**, 236-240 (1999).
30 Kumari, R., Kumar, R. & Lynn, A. g_mmpbsa—A GROMACS Tool for High-Throughput MM-PBSA Calculations. *Journal of Chemical Information and Modeling* **54**, 1951-1962 (2014).
31 Kollman, P. A. *et al.* Calculating structures and free energies of complex molecules: combining molecular mechanics and continuum models. *Accounts of chemical research* **33**, 889-897 (2000).
32 Wagoner, J. A. & Baker, N. A. Assessing implicit models for nonpolar mean solvation forces: the importance of dispersion and volume terms. *Proc Natl Acad Sci USA* **103**, 8331-8336 (2006).

33 Nag, S. *et al.* Nature of the Amyloid-beta Monomer and the Monomer-Oligomer Equilibrium. *Journal of Biological Chemistry* **286**, 13827-13833 (2011).
